# Brain Punch: K-1 Fights Affect Brain Wave Activity in Professional Kickboxers

**DOI:** 10.1007/s40279-024-02082-5

**Published:** 2024-08-07

**Authors:** Łukasz Rydzik, Marta Kopańska, Wojciech Wąsacz, Ibrahim Ouergui, Zbigniew Obmiński, Tomasz Pałka, Tadeusz Ambroży, Nikos Malliaropoulos, Nicola Maffulli, Kabir Singh Lota, Jarosław Jaszczur-Nowicki, Paweł Król, Wojciech Czarny, Jacek Szczygielski

**Affiliations:** 1 Institute of Sports Sciences, University of Physical Education, al. Jana Pawła II 78, 31-571 Kraków, Poland; 2 Department of Pathophysiology, Institute of Medical Sciences, Medical College of Rzeszów University, 35-959 Rzeszów, Poland; 3 High Institute of Sport and Physical Education of Kef, University of Jendouba, 7100 El Kef, Tunisia; 4 Department of Endocrinology, Institute of Sport-National Research Institute, 01‐982 Warsaw, Poland; 5 Department of Physiology and Biochemistry, Faculty of Physical Education and Sport, University of Physical Education, 31-571 Krakow, Poland; 6 Centre for Sports and Exercise Medicine, Queen Mary University of London, London, E1 4DG UK; 7 Sports and Exercise Medicine Clinic, 54639 Thessaloniki, Greece; 8 Sports Clinic, Rheumatology Department, Barts Health NHS Trust, London, E1 4DG UK; 9 Department of Trauma and Orthopaedic Surgery, School of Medicine and Psychology, La Sapienza University, Rome, Italy; 10 Institute of Science and Technology in Medicine, Keele University School of Medicine, Keele University, Newcastle-under-Lyme, UK; 11 Department Physiotherapy, School of Public Health, Collegium Medicum, University of Warmia and Mazury, 10-719 Olsztyn, Poland; 12 Institute of Physical Culture Studies, College of Medical Sciences, University of Rzeszow, 35-959 Rzeszów, Poland; 13 Department of Neurosurgery, Institute of Medical Sciences, University of Rzeszów, 35-959 Rzeszów, Poland; 14 Department of Neurosurgery, Saarland University and Saarland University Medical Center, Homburg, Saarland Germany

## Abstract

**Background:**

Kickboxing is a popular striking combat sport, and K-1 is a type of kickboxing. Direct head blows can cause significant long-term injury and affect brain wave activity.

**Objectives:**

We aim to compare the changes in brain wave activities of fighters during a K-1 kickboxing contest to those in a control group, who were striking a punching bag and were not hit by another K-1 athlete.

**Methods:**

A total of 100 professional Polish K-1 kickboxers were split evenly into experimental (*n* = 50, age 25.5 ± 4.63 years) and control (*n* = 50, age 26.6 ± 5.22 years) groups. We used quantitative electroencephalography (QEEG) to assess the spectrum of brain wave activity (delta, theta, alpha, sensorimotor rhythm (SMR), beta-1 and beta-2) before and after an intervention (experimental: K-1 contest, control: simulated contest), with eyes open and then closed. The number of direct blows to the head was also recorded for all bouts. Comparative and statistical analyses between selected variables were performed.

**Results:**

K-1 fighters showed elevated baseline brain activity for the entire delta band (*p* < 0.001). There was significant variation in brain activity among the experimental group following the intervention and compared with the control group for all wave types (*p* < 0.001). No significant variation in activity was found in the control group. The number of direct head blows was positively correlated with brain activity, at delta and beta-2 wave frequencies.

**Conclusions:**

K-1 kickboxing is associated with detectable changes in brain wave activity. It is presently unclear what the long-term effects of these changes in brain wave activities are, and longitudinal studies are necessary to study the brain health of kickboxers.

**Supplementary Information:**

The online version contains supplementary material available at 10.1007/s40279-024-02082-5.

## Key Points

**Table:** 

K-1 kickboxing fights are associated with significant changes in brain wave activity, particularly in delta and beta-2 frequencies.
The number of direct head blows during a fight correlates with increased brain activity in specific wave frequencies.
Further research is necessary to understand the long-term effects of these changes on the brain health of kickboxers.

## Introduction

Combat sports enjoy growing popularity but carry a significant risk of injury [1–4]. In sports with an emphasis on striking, the head is particularly vulnerable, and repeated blows can lead to adverse morphological and functional changes within the brain [5, 6].

Kickboxing is an example of a striking combat sport. Bouts are generally contested in a standing position, with players using their upper and lower limbs to impact opponents. It is important to note that kickboxing is not an Olympic sport [7]. The absence of a single international governing body has meant that various styles of kickboxing now exist. K-1 is one of many professional kickboxing promotions operating under its own ruleset. In K-1, permitted striking techniques include punches, sweeps, kicks and knees exclusively, with use of the elbows strictly prohibited. Contests are scored based on number of knockdowns scored, opponent damage, number of strikes and overall aggression. According to the official website of the World Association of Kickboxing Organizations (WAKO) (https://wako.sport/), K-1 rules allow for a variety of striking techniques, making it one of the most physically demanding combat sports. K-1 kickboxing is a striking combat sport that requires a high level of physical and physiological conditioning [8, 9]. It demands significant anaerobic and aerobic capacity, explosive strength, speed, agility and endurance [10]. Athletes must possess quick reflexes, high pain tolerance and the ability to sustain intense physical exertion over multiple rounds.

Injuries from head impacts during kickboxing training and competition can have severe long-term neurological consequences. In fact, the sport has been identified as a newer cause of hypopituitarism, secondary to traumatic brain injury [11, 12]. Kickboxers can receive a high number of direct head strikes during a single contest [13]. The knowledge and behaviours of players and coaches regarding brain injury in kickboxing has also been investigated, but there remains a paucity of relevant research in the sport [14, 16]. It was recently reported that K-1 fighters—during the off-training period—exhibit brain wave patterns which can have a negative effect on athletic performance, and possibly make players more susceptible to injury [16, 17]. Additionally, non-standard brain activities have been demonstrated both immediately before a fight and in situations associated with coping, stress, arousal or concentration, which may also increase the risk of brain injury [18]. Among them, the important indicator of the potentially pathological electrical brain activity is the change in pattern of synchronization/desynchronization of brain waves. The terms synchronization and desynchronization describe changes in the frequency of brain waves, potentially accessed by electroencephalography (EEG). Since brain waves represent the neuronal activity in the brain, synchronization and desynchronization may reflect some aspects of functional architecture of this activity [19, 20]. Coordination and integration of different brain areas results in synchronization, i.e. these distinct parts of the brain generate waves of similar pattern and frequency/oscillation. Under this condition, the information processing may be better coordinated [21, 22].

Desynchronization describes the lack of such coordination between different brain areas. On one hand, this phenomenon may accompany regular sensorimotor activity or may result from the more autonomous activity of highly specialized parts of the brain. This may be advantageous for information processing, as occasionally reported in assessment of meditative states or situations, to which the high level of psychological concentration is attributed [23–28]. On the other hand, desynchronization may occur in diverse neurological and psychiatric conditions, including epilepsy [29, 30] or neurodegenerative disorders [31–33]. Thus, their analysis may be helpful in diagnostic and therapeutic workup of these conditions. From the vantage point of combat sport medicine, the changes in quantitative electroencephalography (QEEG) activity, including synchronization and desynchronization, may result from the mental focus and motor activity of a fighter during a bout [34, 35] or from the disturbance caused by repetitive mild injuries to the brain [23, 36]. To date, it remains unclear to what extent K-1 fights alter brain activity. QEEG, otherwise known as ‘brain mapping’, is an abbreviated version of EEG that measures electrical activity in the form of brainwave patterns. The application of this diagnostic tool has been validated in competitive sport and utilised in similar kickboxing research [16, 17, 37]. Therefore, the aim of the present study was to compare changes in the brain wave activities of fighters during a K-1 kickboxing contest with those in a control group, who were striking a punching bag and were not hit by another K-1 athlete. We hypothesized that significant differences in brain activity profile would be present between these two populations.

## Methods

The study was conducted in accordance with the Declaration of Helsinki and approved by the Ethics Committee at the University of Rzeszów (protocol code: 2022/038).

### Participants

Professional K-1 kickboxers based in Poland were eligible for participation in this study. The inclusion criteria were at least 5 years kickboxing experience, injury-free, valid medical check-up certificate, no history of heavy knockouts and active participation in competition. A minimum sample size of 100 was calculated using G*Power.

A total of 100 K-1 kickboxers were recruited and distributed evenly between experimental (*n* = 50) and control (*n* = 50) groups. The mean age (years), height (cm), weight (kg) and body mass index (BMI) (kg/m^2^) of participants in the experimental group was 25.5 ± 4.6 (range 18–33) years, 179.3 ± 6.1 (range 168–189) cm, 76.7 ± 10.0 (range 61.4–94.1) kg and 23.8 ± 2.5 kg/m^2^, respectively. The mean age (years), height (cm), weight (kg) and BMI (kg/m^2^) of participants in the control group was 26.6 ± 5.2 (range 18–35) years, 177.7 ± 5.6 (range 165–187) cm, 77.9 ± 7.5 (range 65.7–90.0) kg and 24.7 ± 2.1 kg/m^2^. respectively. The minimum training experience of all participants was 5 years. Fighters were then given a unique identification number and randomly assigned to the experimental or control group, using a random number generator. All participants provided informed written consent prior to testing.

### Data Collection

Participants were assigned to the following weight categories: < 65 kg, < 71 kg, < 76 kg, < 81 kg, < 86 kg, < 91 kg and > 91 kg. Each participant wore 10 oz boxing gloves, foot and shin guards and no protective headgear. They were also asked not to consume caffeine, energy drinks or any dietary supplementary 24 h before testing and asked to record their eating habits in the 14 days prior to testing, using the Fitatu app (Dietis, Poland) on their smartphones. Finally, they were advised to avoid sparring in the week before testing and to avoid any significant physical activity in the 48 h before testing. All data were collected between 15 May 2022 and 25 June 2022.

In the experimental group, brain activity of players was measured whilst warming up for a professional K-1 kickboxing bout and in the immediate 3 min after completion of the bout. Each bout was contested over three 2-min rounds, with a 1-min break between rounds, in accordance with the rules of the World Association of Kickboxing Organizations (WAKO). Fights were recorded using a GoPro HERO 10 camera for further analysis of impacts delivered to the head.

The control group undertook a simulated fight exercise on a punching bag (height 180 cm, weight 80 kg) (Octagon, Poland), over three 2-min periods, with a 1-min break between each period, to mirror the structure of a K-1 bout. The number of blows delivered to the punching bag was supervised and controlled by a member of the research team. All fights were observed by referees, coaches and research co-ordinators.

### Equipment and Procedure

QEEG offers a numeric, spectral analysis of the EEG record, with data being digitally coded and analysed using the Fourier transform algorithm [38]. Testing of a single participant lasted approximately 10–15 min and included two stages: recording of brain wave activity with the eyes open (3 min) and then with the eyes closed (3 min). The wave amplitude and power for specific frequencies were subsequently analysed.

**Fig. 1:**
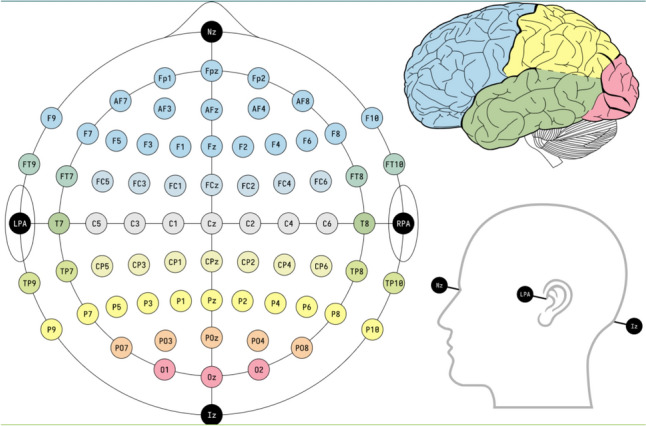
Schematic of the arrangement and range of signal readings from electrodes used during quantitative electroencephalography (QEEG). We analysed the signal from the points Fz, F3, F4, Cz, C3, C4 and Pz, P3, P4

The study measured delta, theta, alpha, sensorimotor rhythm (SMR), beta-1 and beta-2 waves at electrodes on nine landmarks (frontal: Fz, F3, F4, central: Cz, C3, C4, parietal: Pz, P3, P4) (Fig. 1).

**Fig. 2:**
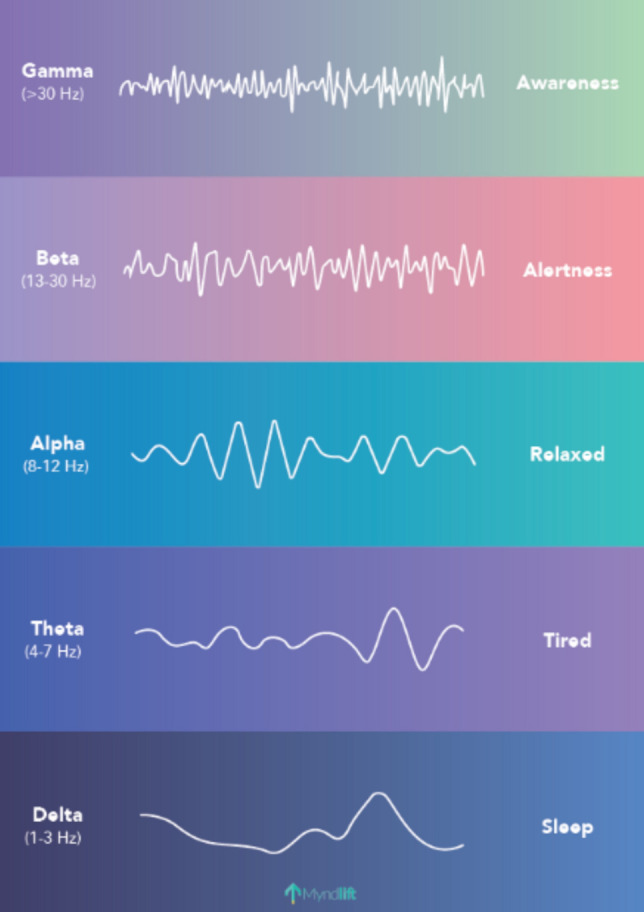
Pattern of typical EEG wave forms as seen in classic EEG recording. Source: https://www.myndlift.com/post/what-are-brainwaves, accessed on 20 Dec 2023, Licence Myndlift Ltd, implemented according to ‘fair use’ principles

Accounting for normal adult values, it is assumed that lower wave frequency is associated with higher wave amplitude. Normal values have been referred to as: delta waves less than 20 µV, theta less than 15 µV, alpha less than 10 µV and SMR, beta 1 and beta 2 all less than 4–10 µV. The typical forms of EEG waves are demonstrated in Fig. 2. Of note, the QEEG output is based on the wave frequency rather than its shape. Therefore, delta and theta bands, for example, may appear in a normal QEEG record, even in the absence of standard forms of delta and theta waves, which is seen in the classic EEG before transformation.

The differentiation between separate wave types is based on the frequency (in Hz); however, the standard interpretation of EEG includes also the subjective assessment of wave shape, while QEEG is based on automated frequency analysis. Thus, even in the absence of the typical form of given waves (e.g. delta), its band may appear in QEEG analysis (e.g. delta wave spectrum).

The EEG signal was transformed using the Cz montage and electrode, with the Elmiko, DigiTrack software (version 14, PL) (ELMIKO, Warsaw, Poland).

The amplitude of QEEG waves was calculated based on the medical standards of the DigiTrack apparatus. The spectrum of a signal is a representation of its frequency. The fast Fourier transform (FFT) algorithm was used with the function: *f*(*z*) = *A*(*z*) + j*F(*z*). The following parameters were used for analysis: minimal signal amplitude of 0.5 µV with minimal temporal distance between single maximal values of 0.5 Hz. Analyses were performed using a computing buffer of 8.2 s (assessment points: 2048; accuracy: 0.12 Hz). Consequently, the set of amplitude values for each part of the frequency spectrum was obtained. The gap between single values measured in Hz defines a calculation resolution. According to the FFT algorithm, this parameter depends on signal sampling frequency and on the length of the computing buffer: *r* = fs/*N*, where *r* is calculation resolution (the gap between single records), fs is the signal sampling frequency and *N* is the length of the computing buffer. The spectrum analysis of the FFT panel in DigiTrack showed peak-to-peak amplitudes. To ensure appropriate reliability, measurement was performed over several seconds [39]. The epoch length determines the frequency resolution of the Fourier transform, with a one-second epoch providing a 1 ± 0.5 Hz resolution and a 4-s epoch providing 0.25 ± 0.125 Hz resolution. The elimination of artifacts from the EEG recording was performed manually and automatically [39]. This protocol was previously tested and validated in pilot research [16, 17].

### Statistical Analysis

All statistical analyses were carried out using the language R (version 4.1.1) [17] on a 64-bit Windows 10 Pro system (19045 compilation), using the following packages: effectsize (version 0.8.3) [40], (version 0.5.29) [41], Hmisc (version 4.7.1) [20], report (version 0.5.7) [42], ggstatsplot (version 0.9.3) [43], lattice (version 0.20.44) [23], gtsummary (version 1.6.2) [44], ggplot2 (version 3.4) [45], readxl (version 1.3.1) [46], dplyr (version 1.1.2) and Formula (version 1.2.4).

Shapiro–Wilk was used to test for normality among numerical variables. These were reported as mean (SD) for normally distributed variables, and as median (Q1, Q3) for variables with non-normal distributions. In the case of normally distributed variables, Student’s *t* test was used with an effect size estimate of Hedges’ *g* [47]. The correlation between the two numerical variables was estimated using Pearson’s linear correlation coefficient. Interpretation of the magnitude of the correlation coefficients was performed using the Funder convention [48]. The Wilcoxon rank sum test was used to test for differences between two independent groups within a variable with a non-normal distribution. An effect size measure was reported as ${\widehat{r} }_{\text{biserial}}^{\text{rank}}$ [49]. In the case of a normally distributed variable, Welch’s *t* test was used with an effect size estimate of Hedges’ *g*. Testing for differences between two dependent groups within a numerical variable with a non-normal distribution was performed using the Wilcoxon signed-rank test. An effect size measure was reported as ${\widehat{r} }_{\text{biserial}}^{\text{rank}}$. Significance was set at *α* = 0.05 for all statistical testing.

## Results

Detailed descriptive statistics for all results are provided in the Supplementary Material Appendices A-I.

### Effect of the Intervention in the Experimental Group

**Fig. 3:**
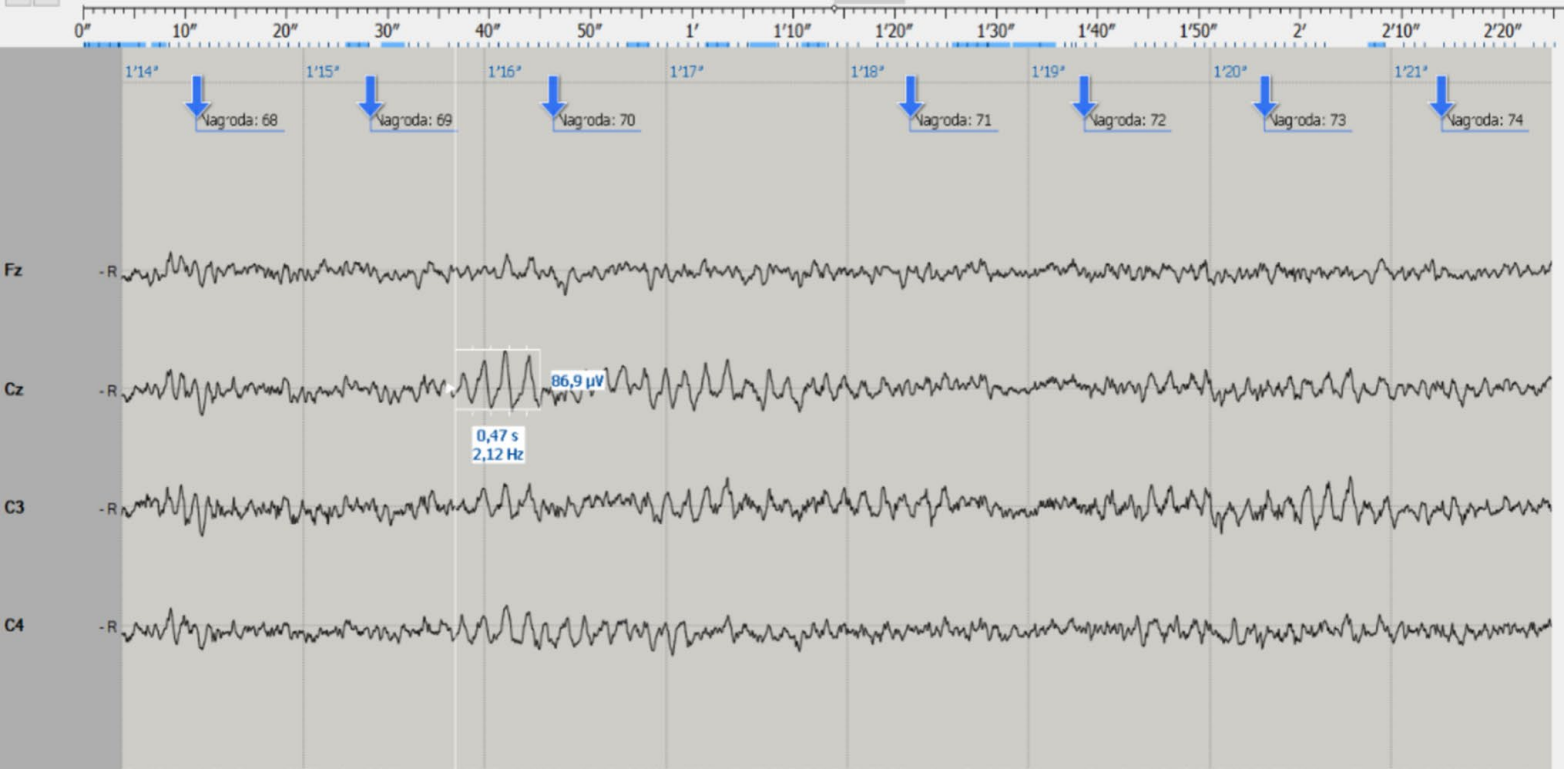
Example of the EEG record before the kickboxing fight. The image represents the step before the transformation to quantitative electroencephalography (QEEG) analysis. Here, the region of interest (ROI) frame visualizes the principle of quantitative assessment of both frequency and amplitude of the EEG waves

**Fig. 4:**
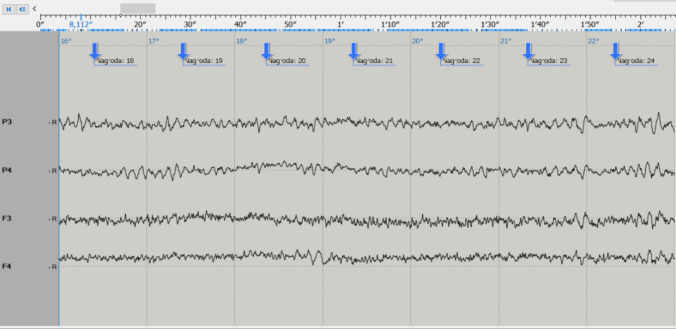
Example of the EEG record after the kickboxing fight. As in Fig. 3, the EEG record represents the step before the transformation to quantitative electroencephalography (QEEG) analysis. Note the increased diversity of the record with the increased beta-type activity and several slow waves

In the experimental group, significant differences were found between the values of 40 (74%) QEEG parameters, with the eyes open. The examples of the raw records before the conversion to quantitative analysis (QEEG) are demonstrated in Figs. 3 and 4.

Significant increases in activity were demonstrated in 38 parameters, with significant decreases detected in the other two. According to wave types, these parameters included: delta waves: *n* = 7 (78%), theta waves: *n* = 7 (78%), alpha waves: *n* = 5 (56%), SMR waves: *n* = 8 (89%), beta-1 waves: *n* = 7 (78%) and beta-2 waves: *n* = 6 (67%). According to electrode positions, the number of the significant parameters included: Fz: *n* = 5 (83%), F3: *n* = 5 (83%), F4: *n* = 3 (50%), Cz: *n* = 4 (67%), C3: *n* = 4 (67%), C4: *n* = 2 (33%), Pz: *n* = 6 (100%), P3: *n* = 5 (83%) and P4: *n* = 6 (100%).

Significant differences were also detected between the values of 42 (78%) QEEG parameters, with the eyes closed. Significant increases in activity were similarly demonstrated in 38 parameters, with significant decreases noted in the other four. According to wave types, these parameters included: delta waves: *n* = 6 (67%), theta waves: *n* = 6 (67%), alpha waves: *n* = 9 (100%), SMR waves: *n* = 8 (89%), beta-1 waves: *n* = 7 (78%) and beta-2 waves: *n* = 6 (67%). According to electrode positions, the number of the significant parameters included: Fz: *n* = 4 (67%), F3: *n* = 5 (83%), F4: *n* = 4 (67%), Cz: *n* = 5 (83%), C3: *n* = 5 (83%), C4: *n* = 2 (33%), Pz: *n* = 6 (100%), P3: *n* = 5 (83%), and P4: *n* = 6 (100%).

### Effect of the Intervention in the Control Group

In the control group, there was no statistically significant difference between measurements before and after testing, with the eyes open. However, a significant decrease in post-exercise activity was detected in control group participants for beta-1 15–20 Hz waves (C3), with the eyes closed. There were no other significant differences before and after testing.

### Pre-fight QEEG Measurements Between Groups

Pre-fight examinations with the eyes open showed significant differences in 48 (89%) QEEG parameters between experimental (*n* = 23) and control (*n* = 25) groups. According to wave types, these parameters included: delta waves: *n* = 9 (100%), theta waves: *n* = 9 (100%), alpha waves: *n* = 7 (78%), SMR waves: *n* = 8 (89%), beta-1 waves: *n* = 6 (67%) and beta-2 waves: *n* = 9 (100%). According to electrode positions, the number of the significant parameters included: Fz: *n* = 6 (100%), F3: *n* = 6 (100%), F4: *n* = 5 (83%), Cz: *n* = 6 (100%), C3: *n* = 6 (100%), C4: *n* = 5 (83%), Pz: *n* = 5 (83%), P3: *n* = 4 (67%) and P4: *n* = 5 (83%).

**Fig. 5:**
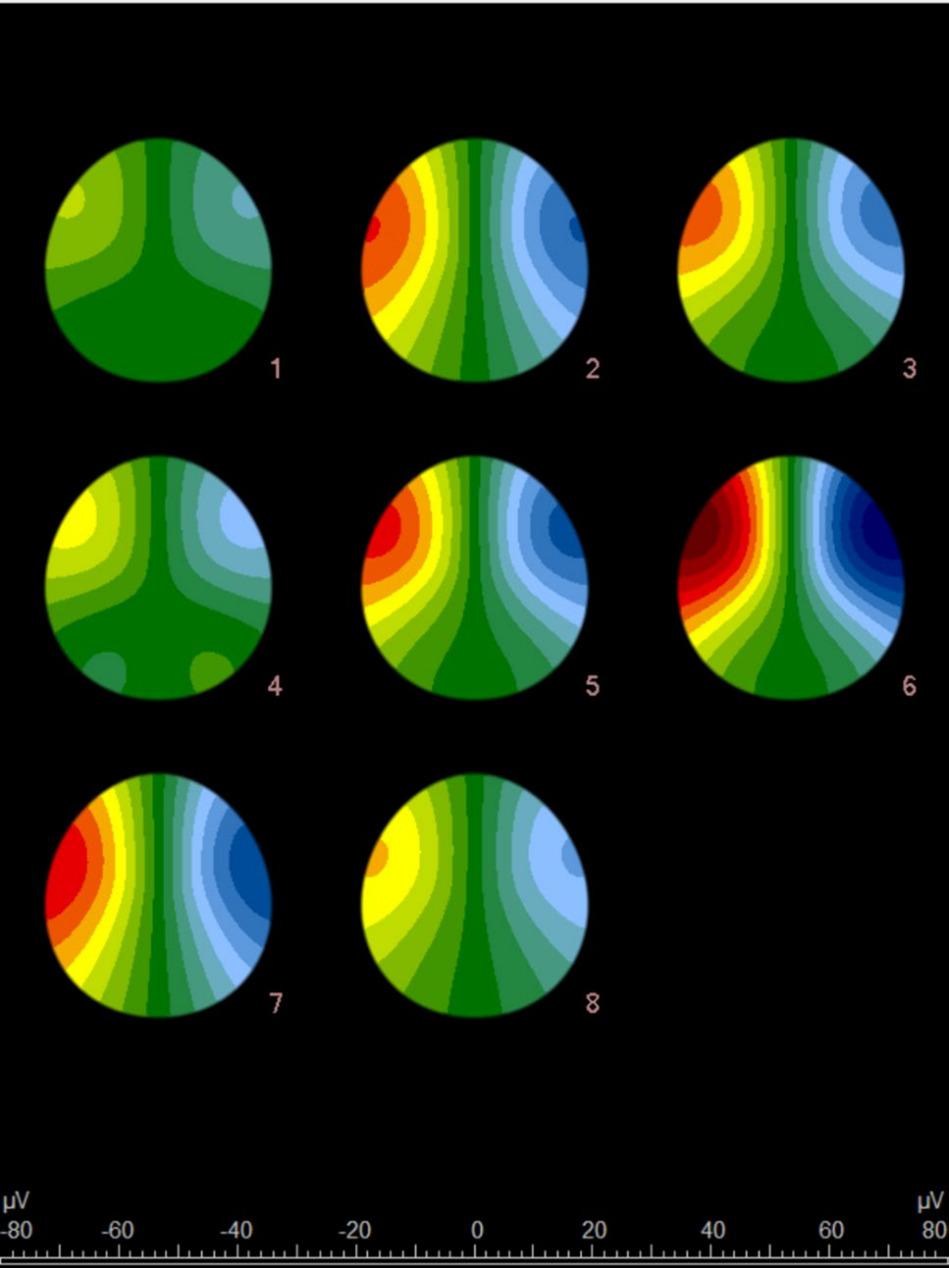
Quantitative electroencephalography (QEEG) brain mapping analysis method showing the distribution of brain wave activity of different frequencies across the scalp of a kickboxer. The findings are based on the different amplitudes of the QEEG waves. A non-homogeneous amplitude distribution is found, resulting mostly from the position of the electrodes in relation to the reference. The maps illustrate the distribution of brain wave activity before and after the fight under different conditions: (1) baseline (pre-fight) with eyes open, (2) baseline (pre-fight) with eyes closed, (3) immediate post-fight with eyes open, (4) immediate post-fight with eyes closed, (5) 3 min post-fight with eyes open, (6) 3 min post-fight with eyes closed, (7) 5 min post-fight with eyes open and (8) 5 min post-fight with eyes closed. Warmer colours (red) in the figure indicate higher brain wave activity (measured in μV) and cooler colours (blue/green) indicate lower activity

Pre-fight examinations with the eyes closed showed significant differences in 40 (74%) QEEG parameters between experimental (*n* = 17) and control (*n* = 23) groups. According to wave types, these parameters included: delta waves: *n* = 9 (100%), theta waves: *n* = 8 (89%), alpha waves: *n* = 6 (67%), SMR waves: *n* = 6 (67%), beta-1 waves: *n* = 5 (56%) and beta-2 waves: *n* = 6 (67%). According to electrode positions, the number of the significant parameters included: Fz: *n* = 5 (83%), F3: *n* = 5 (83%), F4: *n* = 3 (50%), Cz: *n* = 3 (50%), C3: *n* = 5 (83%), C4: *n* = 6 (100%), Pz: *n* = 5 (83%), P3: *n* = 4 (67%) and P4: *n* = 4 (67%). Detailed descriptive statistics and graphical representation of the results are provided in Supplementary Material Appendices A-I. Figure 5 demonstrates a potential of quantitative EEG analysis with visualized QEEG amplitude in both hemispheres. These QEEG brain maps show notable increases in brain wave activity, particularly in delta and beta-2 frequencies, in the frontal and occipital regions, demonstrating the effect of head impacts during the fight.

These QEEG brain maps show notable increases in brain wave activity, particularly in delta and beta-2 frequencies, in the frontal and occipital regions, demonstrating the effects of head impacts during the fight.

### Post-fight QEEG Measurements Between Groups

Post-fight examinations with the eyes open showed significant differences in 44 (81%) QEEG parameters between experimental (*n* = 38) and control (*n* = 6) groups. According to wave types, these parameters included: delta waves: *n* = 8 (89%), theta waves: *n* = 9 (100%), alpha waves: *n* = 9 (100%), SMR waves: *n* = 5 (56%), beta-1 waves: *n* = 4 (44%) and beta-2 waves: *n* = 9 (100%). According to electrode positions, the number of the significant parameters included: Fz: *n* = 5 (83%), F3: *n* = 5 (83%), F4: *n* = 4 (67%), Cz: *n* = 4 (67%), C3: *n* = 4 (67%), C4: *n* = 6 (100%), Pz: *n* = 6 (100%), P3: *n* = 5(83%) and P4: *n* = 5 (83%).

Post-fight examinations with the eyes closed showed significant differences in 42 (78%) QEEG parameters between experimental (*n* = 34) and control (*n* = 8) groups. According to wave types, these parameters included: delta waves: *n* = 6 (67%), theta waves: *n* = 8 (89%), alpha waves: *n* = 9 (100%), SMR waves: *n* = 4 (44%), beta-1 waves: *n* = 7 (78%) and beta-2 waves: *n* = 8 (89%). According to electrode positions, the number of the significant parameters included: Fz: *n* = 3 (50%), F3: *n* = 5 (83%), F4: *n* = 3 (50%), Cz: *n* = 6 (100%), C3: *n* = 6 (100%), C4: *n* = 4 (67%), Pz: *n* = 5 (83%), P3: *n* = 5(83%) and P4: *n* = 5 (83%).

### Effect of Direct Head Blows on QEEG Measurements

The median number of direct head blows was 48.0 (Q1 is 41.0, Q3 is 56.0). This was significantly correlated with seven QEEG parameters. An increase in the number of head impacts was associated with an increase in delta (0.5–4 Hz) (Fz, C3, C4, Pz, P3, P4) and beta-2 (20–35 Hz) (Cz) wave activity.

## Discussion

Characterisation of the QEEG parameters for testing with the eyes open and closed revealed that, before the application of the experimental stimulus, baseline brain activity was elevated amongst the entire cohort. This was most clearly observed examining delta frequencies in the frontal band (Fz, F3, F4), with the eyes open. These results exceeded values seen in the normal condition/in individuals not affected by any neurological or psychiatric condition as a regular component of the global QEEG spectrum [50–53], or were closer to the upper limit of these reference norms. Higher percentage of delta amplitudes and frequencies in kickboxers could be associated with various neurological and psychiatric problems, including chronic traumatic encephalopathy (CTE) [54, 55]. Non-traumatic forms of cognitive decline were also associated with increased percentages of delta activity in spectral analysis, as highlighted in previous reports [50, 56, 57]. Such changes may occur because of the long-term training and competitive activities of the participants, and direct head blows can damage brain structures responsible for generating brain waves [11, 58]. Post-traumatic changes in metabolic function or perfusion of affected areas can also be postulated, based on studies implementing both QEEG and brain perfusion analysis [59–62]. Other mechanisms beside direct head blows may also be responsible for the recorded changes. Defensive actions in kickboxing offer immediate protection for athletes but indirectly transmit vibrations to the brain. These are also capable of causing structural or metabolic changes in the brain, and it is vital that above-average delta wave activity receives further medical investigation and is potentially taken into account in therapeutic strategies [63].

Our analyses illustrated significant alterations in brain activity in the experimental group both with open and closed eyes, which represent different circumstances regarding brain function. With the eyes open, QEEG measures brain activity during the reception of external stimuli involving vision or its synergy with other senses. The work of the brain is focused on processing external information (activation of cognitive processes and sensory perception), while the power of amplitudes can increase after performing, for example, a complex motor task [64]. The eyes-closed variant, on the other hand, eliminates the perception of the external environment, which may promote intrinsic brain activities, that are directed towards rest, recovery, relaxation and meditation. These processes are typically conducive to lower frequency waves, whereas increased frequency is often associated with creativity, focus, alertness and concentration [16, 65]. Here, changes in brain activity were noted also at lower frequencies. This was especially the case for the delta wave part of the spectrum (0.5–4 Hz), in the frontal (Fz, F3, F4) and occipital (Pz, P3, P4) bands, with the eyes open, which significantly exceeded normal values. A similar trend was also observed for examinations with the eyes closed. Here, we postulate that these elevated baseline activities may reflect years of kickboxing training and competition in general. In turn, observed changes in experimental activity from baseline are likely due to the contest fought by participants for the purpose of the study, as combat sport competition is linked to increased concentration and alertness [66, 67].

More intense brain activities were observed in the experimental group for all other brain wave types (theta, alpha, SMR, beta-1, beta-2) with eyes both open and closed. Again, these were likely induced by the study stimulus. In the context of kickboxing, increases in theta band activity represent a state of arousal, inner focus and fight readiness, all of which would have also been experienced in previous fights [68]. The higher theta amplitudes in the frontal-medial line may be associated with improved performance in professional athletes [69]. Increased alpha wave frequencies were generally observed with eyes closed (also known as alpha rhythm), for all measurement bands, indicating a state of relaxation after completion of a task [70]. During competition, kickboxers are exposed to high levels of stress and exertion, resulting in both physical and mental tension. When a fighter closes their eyes, he or she prevents the sense of vision from recording external stimuli. This can help to reduce tension, thereby increasing alpha wave activity in the brain, and is thought to benefit recovery and general wellbeing [71]. SMR waves are primarily generated in the sensorimotor regions of the brain and are associated with motor cortex activation. Increased activity, as observed in our data, may reflect the high levels of motor coordination and concentration during a contest, as well as the state of relaxation after. SMR activity levels are positively correlated with the ability of athletes to control their physiological responses, such as heart rate and breathing, and help achieve a desired state of mind [72, 73]. The activity of beta-1 waves, similarly to delta waves, is thought to be related to the number of blows received to the head [13]. Beta-2 waves work to regulate emotions and stress responses, and higher activities might be explained by participants actively counteracting the anxieties of an upcoming fight or the analytical thinking and planning of performance, where decisiveness and the ability to make quick decisions are necessary [74–76].

In contrast, comparison of brain activity in the control group before and after a simulated bout showed no significant differences in the parameters evaluated. This suggests that although isolated kickboxing training that excludes opponent confrontation is a stressful stimulus, this stress is rather physical and does not directly cause any significant changes in brain wave activity. Furthermore, comparative analyses between the experimental and control groups revealed a diverse profile of brain activities induced by the intervention stimulus. Interestingly, there was a significantly higher proportion of alpha waves in the experimental group for both variants of testing at each bandwidth. This may imply that the situation where opponents are directly facing one another causes a cross-sectional physical and emotional discharge of the fighters. Consequently, the intensity of alpha wave amplitudes is promoted, precipitating a state of relaxation after a contest. Finally, many researchers argue that exposure to stress increases brain wave activity [77–79]. Therefore, it appears reasonable to suggest that the stress faced by athletes in a real contest constitutes higher wave activities [80]. Importantly, changes in brain activity may be influenced by the quantity and quality of blows received to the head. Indeed, this is confirmed by our data, where a significant correlation was found between the number of direct head blows and delta (Fz, C3, C4, Pz, P3, P4) and beta-2 (Cz) wave activity. This is consistent with previous observations, linking the metabolic impairment and changes of electrophysiologic function of the brain with even mild but repetitive traumatic brain injury [61, 62, 81]. This highlights both the importance of the recorded changes for precipitation of the chronic posttraumatic encephalopathy in kickboxers and the potential usefulness of QEEG to monitor these changes and—if applicable—the efficacy of therapeutic interventions [82–84].

### Limitations

This study has some limitations. For example, we did not perform imaging to ascertain whether brain morphology was within normal limits. Also, we did not stratify for training and competition age. Our control group was composed of equally trained K-1 fighters with potential impacts from previous bouts: non-athletes, or athletes involved in fighting sports in which striking is not allowed, such as wrestling, judo, or Brazilian jiu-jitsu, could have been enrolled. However, within the inevitable limits of our experimental set up, we recruited a statistically significant number of K-1 fighters, the procedures were performed following a strict protocol, and the results obtained are scientifically valid. Furthermore, by comparing the QEEG records in these highly standardized conditions, using two homogeneous and very similar groups, we believe that our results demonstrate the possible early impact of the single series of events, including repetitive blows to the head, that accompany each kickboxing fight.

## Conclusions

K-1 kickboxing contests can cause significant changes in brain wave activity. This study helps to understand the effect of kickboxing on physical and mental function and how to protect the health of its participants. With the correct training methods, kickboxing can be safely practiced by those who do not wish to make the sport their profession, which requires direct confrontation with sparring partners and opponents.

## Supplementary Information

Below is the link to the electronic supplementary material.

Supplementary file1 (DOCX 214 KB)

## Declarations

### Data availability statement

All data have been included in the manuscript and its Supplementary Materials.
