# Supplementary material for: Brain Punch: K-1 Fights Affect Brain Wave Activity in Professional Kickboxers
Source: Sports Med. 2024 Aug 7;54(12):3169–79. doi: 10.1007/s40279-024-02082-5 (PMC11608281; doi:10.1007/s40279-024-02082-5)
Supplement: Supplementary file 1 — Supplementary file1 (DOCX 214 KB) [file 40279_2024_2082_MOESM1_ESM.docx]

**Supplementary material**

**Appendix A**. Effect of the intervention in the experimental group with eyes open

| *QEEG parameter* | *Time point* | | *p-value^3^* |
| --- | --- | --- | --- |
|  | *pre-fight, n = 50^1^* | *post-fight, n = 50^1^* |  |
| Delta 0.5Hz-4Hz (Fz) | 20.13 (19.85, 20.83) | 28.99 (25.60, 32.85) | **<0.001** |
| Delta 0.5Hz-4Hz (F3) | 22.02 (21.06, 23.77) | 26.74 (25.49, 28.15) | **<0.001** |
| Delta 0.5Hz-4Hz (F4) | 21.55 (20.46, 22.55) | 26.07 (25.10, 26.59) | **<0.001** |
| Delta 0.5Hz-4Hz (Cz) | 14.35 (13.25, 15.57) | 15.57 (15.36, 19.24) | **0.001** |
| Delta 0.5Hz-4Hz (C3) | 13.69 (13.32, 16.69) | 13.80 (13.21, 16.52) | 0.980 |
| Delta 0.5Hz-4Hz (C4) | 13.61 (12.05, 17.70) | 13.71 (13.14, 16.72) | 0.582 |
| Delta 0.5Hz-4Hz (Pz) | 11.51 (10.81, 12.64) | 36.98 (35.69, 38.77) | **<0.001** |
| Delta 0.5Hz-4Hz (P3) | 11.59 (11.21, 12.64) | 36.34 (31.70, 38.36) | **<0.001** |
| Delta 0.5Hz-4Hz (P4) | 9.23 (8.79, 9.87) | 36.92 (35.67, 37.88) | **<0.001** |
| Theta 4Hz-8Hz (Fz) | 12.39 (11.25, 14.81) | 15.78 (14.99, 16.98) | **<0.001** |
| Theta 4Hz-8Hz (F3) | 11.78 (10.78, 12.26) | 12.98 (10.92, 14.22) | 0.349 |
| Theta 4Hz-8Hz (F4) | 12.06 (10.97, 13.91) | 13.72 (9.01, 16.93) | 0.332 |
| Theta 4Hz-8Hz (Cz) | 7.44 (6.94, 8.51) | 8.55 (8.13, 9.03) | **0.008** |
| Theta 4Hz-8Hz (C3) | 7.43 (6.77, 7.64) | 8.02 (7.80, 8.60) | **0.006** |
| Theta 4Hz-8Hz (C4) | 6.58 (5.78, 6.96) | 7.21 (6.40, 8.16) | **0.019** |
| Theta 4Hz-8Hz (Pz) | 6.48 (5.25, 6.98) | 11.32 (8.04, 13.44) | **<0.001** |
| Theta 4Hz-8Hz (P3) | 6.54 (6.01, 6.74) | 10.42 (7.86, 13.51) | **<0.001** |
| Theta 4Hz-8Hz (P4) | 5.25 (5.11, 6.10) | 10.61 (6.80, 12.03) | **<0.001** |
| Alpha 8Hz-12Hz (Fz) | 6.75 (6.42, 7.39) | 7.18 (6.39, 8.12) | 0.540 |
| Alpha 8Hz-12Hz (F3) | 6.56 (5.65, 6.99) | 7.94 (6.38, 11.18) | **<0.001** |
| Alpha 8Hz-12Hz (F4) | 9.50 (6.11, 11.54) | 7.98 (5.48, 10.28) | **0.028** |
| Alpha 8Hz-12Hz (Cz) | 7.53 (5.66, 9.17) | 8.58 (5.71, 10.12) | 0.089 |
| Alpha 8Hz-12Hz (C3) | 7.92 (5.71, 8.79) | 8.59 (6.32, 9.03) | 0.196 |
| Alpha 8Hz-12Hz (C4) | 6.96 (4.88, 7.14) | 6.86 (5.21, 7.37) | 0.657 |
| Alpha 8Hz-12Hz (Pz) | 6.18 (5.32, 8.25) | 9.93 (9.53, 12.62) | **<0.001** |
| Alpha 8Hz-12Hz (P3) | 7.80 (6.11, 8.71) | 8.04 (7.91, 9.03) | **0.018** |
| Alpha 8Hz-12Hz (P4) | 7.08 (4.75, 7.59) | 6.61 (6.39, 8.38) | **0.042** |
| SMR 12Hz-15Hz (Fz) | 4.05 (3.79, 4.61) | 4.92 (4.29, 5.54) | **<0.001** |
| SMR 12Hz-15Hz (F3) | 4.14 (3.83, 5.11) | 6.04 (5.30, 7.01) | **<0.001** |
| SMR 12Hz-15Hz (F4) | 5.07 (3.58, 5.25) | 5.31 (4.22, 6.29) | **0.032** |
| SMR 12Hz-15Hz (Cz) | 4.16 (3.82, 5.17) | 5.48 (4.51, 6.18) | **<0.001** |
| SMR 12Hz-15Hz (C3) | 4.40 (3.81, 5.53) | 6.03 (4.83, 7.24) | **<0.001** |
| SMR 12Hz-15Hz (C4) | 4.40 (3.49, 4.91) | 4.60 (4.25, 5.16) | **0.012** |
| SMR 12Hz-15Hz (Pz) | 4.86 (4.39, 5.76) | 7.26 (6.11, 8.90) | **<0.001** |
| SMR 12Hz-15Hz (P3) | 5.08 (4.45, 5.85) | 5.56 (4.79, 6.77) | 0.054 |
| SMR 12Hz-15Hz (P4) | 4.38 (3.58, 5.44) | 5.23 (4.42, 6.53) | **0.007** |
| Beta-1 15Hz-20Hz (Fz) | 4.95 (4.58, 5.11) | 5.58 (4.96, 5.73) | **<0.001** |
| Beta-1 15Hz-20Hz (F3) | 5.60 (4.98, 5.85) | 6.64 (5.64, 7.28) | **<0.001** |
| Beta-1 15Hz-20Hz (F4) | 6.40 (6.21, 7.32) | 6.68 (6.29, 7.05) | 0.457 |
| Beta-1 15Hz-20Hz (Cz) | 4.81 (4.61, 5.20) | 5.97 (5.42, 6.61) | **<0.001** |
| Beta-1 15Hz-20Hz (C3) | 5.38 (4.86, 5.78) | 6.19 (5.56, 7.14) | **<0.001** |
| Beta-1 15Hz-20Hz (C4) | 5.03 (4.82, 5.27) | 5.23 (4.44, 5.71) | 0.062 |
| Beta-1 15Hz-20Hz (Pz) | 4.82 (4.43, 5.43) | 6.33 (5.66, 7.67) | **<0.001** |
| Beta-1 15Hz-20Hz (P3) | 5.44 (4.93, 5.94) | 6.48 (5.96, 7.02) | **<0.001** |
| Beta-1 15Hz-20Hz (P4) | 5.00 (4.44, 5.43) | 5.81 (5.17, 7.13) | **<0.001** |
| Beta-2 20Hz-35Hz (Fz) | 5.76 (5.20, 6.31) | 6.36 (5.75, 8.12) | **<0.001** |
| Beta-2 20Hz-35Hz (F3) | 7.78 (7.31, 8.47) | 9.84 (7.57, 11.40) | **0.001** |
| Beta-2 20Hz-35Hz (F4) | 9.26 (7.08, 9.39) | 9.47 (7.41, 10.52) | 0.073 |
| Beta-2 20Hz-35Hz (Cz) | 6.72 (6.25, 7.42) | 6.94 (6.55, 8.05) | 0.277 |
| Beta-2 20Hz-35Hz (C3) | 6.40 (6.14, 7.24) | 7.92 (6.74, 8.32) | **<0.001** |
| Beta-2 20Hz-35Hz (C4) | 6.69 (6.38, 7.70) | 6.70 (5.72, 10.06) | 0.220 |
| Beta-2 20Hz-35Hz (Pz) | 6.72 (6.38, 7.49) | 8.09 (7.14, 9.53) | **<0.001** |
| Beta-2 20Hz-35Hz (P3) | 7.10 (7.08, 8.11) | 8.43 (8.14, 9.85) | **<0.001** |
| Beta-2 20Hz-35Hz (P4) | 6.76 (6.07, 7.49) | 9.05 (8.36, 9.11) | **<0.001** |
| *^1^* *Mdn-Median* (Q1-the first quartile , Q3-the third quartile)  *^2^* *M-Mean*  (*SD-Standard Deviation*) | | | |
| *^3^* Wilcoxon rank-sum test with continuity correction  *^4^* Paired Welch's t-test | | | |

**Appendix B**. Effect of the intervention in the experimental group with eyes closed

| *QEEG parameter* | *Time point* | | *p-value^2^* |  |
| --- | --- | --- | --- | --- |
|  | *pre-fight,*  *n = 50^1^* | *post-fight,*  *n = 50^1^* |  |  |
| Delta 0.5Hz-4Hz (Fz) | 16.30 (13.84, 27.33) | 20.77 (17.66, 23.98) | 0.111 |  |
| Delta 0.5Hz-4Hz (F3) | 12.83 (12.19, 14.21) | 22.33 (13.40, 32.70) | **<0.001** |  |
| Delta 0.5Hz-4Hz (F4) | 13.94 (12.28, 16.00) | 21.00 (12.20, 28.29) | **<0.001** |  |
| Delta 0.5Hz-4Hz (Cz) | 16.00 (14.98, 19.45) | 14.86 (13.39, 15.66) | **0.001** |  |
| Delta 0.5Hz-4Hz (C3) | 14.23 (13.98, 16.30) | 16.45 (13.03, 17.44) | 0.862 |  |
| Delta 0.5Hz-4Hz (C4) | 14.63 (13.28, 16.00) | 12.55 (11.07, 17.12) | 0.259 |  |
| Delta 0.5Hz-4Hz (Pz) | 13.94 (12.28, 16.00) | 21.30 (19.33, 29.81) | **<0.001** |  |
| Delta 0.5Hz-4Hz (P3) | 13.03 (12.83, 19.57) | 25.85 (21.30, 26.73) | **<0.001** |  |
| Delta 0.5Hz-4Hz (P4) | 14.31 (13.28, 15.75) | 25.71 (18.16, 26.56) | **<0.001** |  |
| Theta 4Hz-8Hz (Fz) | 14.52 (11.77, 14.91) | 11.80 (9.20, 26.71) | 0.189 |  |
| Theta 4Hz-8Hz (F3) | 12.26 (11.88, 14.11) | 16.83 (12.89, 17.64) | **<0.001** |  |
| Theta 4Hz-8Hz (F4) | 13.91 (12.14, 14.72) | 16.89 (12.12, 17.74) | **0.030** |  |
| Theta 4Hz-8Hz (Cz) | 8.51 (7.38, 9.78) | 9.00 (7.35, 10.23) | 0.058 |  |
| Theta 4Hz-8Hz (C3) | 7.63 (6.59, 9.07) | 7.96 (6.85, 9.58) | **0.029** |  |
| Theta 4Hz-8Hz (C4) | 6.71 (6.35, 10.35) | 6.67 (5.92, 7.52) | 0.159 |  |
| Theta 4Hz-8Hz (Pz) | 9.66 (7.87, 10.62) | 10.22 (9.05, 10.87) | **0.013** |  |
| Theta 4Hz-8Hz (P3) | 9.93 (8.01, 10.43) | 10.64 (9.59, 11.49) | **0.027** |  |
| Theta 4Hz-8Hz (P4) | 10.08 (8.15, 10.46) | 7.78 (6.36, 8.77) | **<0.001** |  |
| Alpha 8Hz-12Hz (Fz) | 8.62 (6.87, 9.48) | 11.87 (8.40, 12.92) | **0.002** |  |
| Alpha 8Hz-12Hz (F3) | 7.45 (6.77, 9.68) | 14.25 (8.12, 19.49) | **<0.001** |  |
| Alpha 8Hz-12Hz (F4) | 6.88 (6.11, 10.12) | 14.00 (6.67, 21.08) | **<0.001** |  |
| Alpha 8Hz-12Hz (Cz) | 7.53 (5.66, 10.70) | 14.84 (9.25, 19.26) | **<0.001** |  |
| Alpha 8Hz-12Hz (C3) | 8.79 (7.92, 9.71) | 11.38 (8.04, 15.51) | **0.002** |  |
| Alpha 8Hz-12Hz (C4) | 7.84 (7.22, 9.57) | 9.47 (7.25, 11.73) | **0.018** |  |
| Alpha 8Hz-12Hz (Pz) | 8.74 (8.25, 10.98) | 11.96 (10.14, 13.57) | **<0.001** |  |
| Alpha 8Hz-12Hz (P3) | 8.71 (7.68, 11.69) | 10.79 (8.95, 13.76) | **<0.001** |  |
| Alpha 8Hz-12Hz (P4) | 9.68 (7.59, 11.14) | 10.83 (7.24, 11.57) | **0.003** |  |
| SMR 12Hz-15Hz (Fz) | 5.08 (4.41, 5.96) | 5.76 (4.88, 5.82) | **0.011** |  |
| SMR 12Hz-15Hz (F3) | 5.22 (4.12, 6.12) | 6.53 (5.66, 7.53) | **<0.001** |  |
| SMR 12Hz-15Hz (F4) | 5.25 (5.07, 6.96) | 5.84 (5.13, 6.68) | 0.369 |  |
| SMR 12Hz-15Hz (Cz) | 5.39 (4.16, 5.87) | 5.69 (5.11, 7.23) | **0.001** |  |
| SMR 12Hz-15Hz (C3) | 4.59 (4.43, 5.23) | 6.38 (5.43, 7.82) | **<0.001** |  |
| SMR 12Hz-15Hz (C4) | 3.92 (3.74, 4.39) | 5.28 (4.04, 6.21) | **<0.001** |  |
| SMR 12Hz-15Hz (Pz) | 4.45 (3.96, 5.74) | 5.72 (5.03, 7.23) | **<0.001** |  |
| SMR 12Hz-15Hz (P3) | 4.45 (4.39, 5.76) | 5.32 (4.94, 8.90) | **<0.001** |  |
| SMR 12Hz-15Hz (P4) | 4.18 (3.58, 5.66) | 5.01 (4.57, 7.58) | **0.005** |  |
| Beta-1 15Hz-20Hz (Fz) | 4.97 (4.50, 5.11) | 6.12 (5.28, 6.78) | **<0.001** |  |
| Beta-1 15Hz-20Hz (F3) | 5.61 (4.98, 5.92) | 6.71 (5.88, 7.13) | **0.001** |  |
| Beta-1 15Hz-20Hz (F4) | 6.21 (5.58, 6.40) | 6.13 (5.86, 6.43) | 0.369 |  |
| Beta-1 15Hz-20Hz (Cz) | 6.19 (5.34, 6.39) | 6.41 (5.46, 7.26) | **0.008** |  |
| Beta-1 15Hz-20Hz (C3) | 6.39 (5.39, 6.93) | 6.61 (6.33, 8.50) | **<0.001** |  |
| Beta-1 15Hz-20Hz (C4) | 5.45 (5.03, 6.27) | 5.71 (5.39, 5.83) | 0.396 |  |
| Beta-1 15Hz-20Hz (Pz) | 4.48 (4.43, 5.43) | 6.80 (5.81, 7.81) | **<0.001** |  |
| Beta-1 15Hz-20Hz (P3) | 5.68 (5.03, 6.34) | 6.21 (5.79, 6.54) | **0.025** |  |
| Beta-1 15Hz-20Hz (P4) | 4.51 (4.43, 5.43) | 5.63 (5.19, 6.28) | **<0.001** |  |
| Beta-2 20Hz-35Hz (Fz) | 5.68 (5.35, 6.31) | 6.72 (5.90, 7.21) | **<0.001** |  |
| Beta-2 20Hz-35Hz (F3) | 7.78 (6.78, 8.47) | 7.17 (6.92, 7.85) | 0.051 |  |
| Beta-2 20Hz-35Hz (F4) | 8.25 (6.56, 9.39) | 6.96 (6.21, 8.38) | **0.003** |  |
| Beta-2 20Hz-35Hz (Cz) | 6.18 (5.50, 6.72) | 7.26 (6.42, 7.83) | **<0.001** |  |
| Beta-2 20Hz-35Hz (C3) | 6.82 (6.40, 7.48) | 7.12 (6.73, 8.34) | **0.025** |  |
| Beta-2 20Hz-35Hz (C4) | 7.39 (6.69, 7.89) | 6.80 (6.58, 7.93) | 0.325 |  |
| Beta-2 20Hz-35Hz (Pz) | 7.49 (6.76, 7.81) | 7.68 (7.13, 9.13) | **0.002** |  |
| Beta-2 20Hz-35Hz (P3) | 8.10 (7.08, 8.71) | 7.94 (7.50, 8.68) | 0.512 |  |
| Beta-2 20Hz-35Hz (P4) | 8.61 (7.14, 8.71) | 6.99 (6.24, 8.56) | **0.038** |  |
| *^1^* *Mdn* (*Q1*, *Q3*) | | | | |
| *^2^* Wilcoxon rank-sum test with continuity correction | | | | |

**Appendix C**. Effect of the intervention in the control group with eyes open

| *QEEG parameter* | *Time point* | | *p-value^3^* |
| --- | --- | --- | --- |
|  | *pre-fight, n = 50^1^* | *post-fight, n = 50^1^* |  |
| Delta 0.5Hz-4Hz (Fz) | 19.91 (19.52, 20.16) | 19.94 (19.58, 20.21) | 0.352 |
| Delta 0.5Hz-4Hz (F3) | 21.52 (20.48, 22.75) | 21.74 (20.43, 22.93) | 0.574 |
| Delta 0.5Hz-4Hz (F4) | 19.89 (19.56, 20.53) | 20.09 (19.64, 20.61) | 0.139 |
| Delta 0.5Hz-4Hz (Cz) | 17.36 (15.97, 18.58) | 16.58 (15.57, 18.36) | 0.722 |
| Delta 0.5Hz-4Hz (C3) | 18.10 (16.25, 20.06) | 18.10 (16.12, 20.13) | 0.899 |
| Delta 0.5Hz-4Hz (C4) | 16.58 (14.99, 18.33) | 16.54 (14.87, 17.94) | 0.525 |
| Delta 0.5Hz-4Hz (Pz) | 18.10 (15.90, 19.74) | 18.34 (16.12, 20.15) | 0.454 |
| Delta 0.5Hz-4Hz (P3) | 20.04 (19.56, 20.32) | 20.09 (19.59, 20.43) | 0.722 |
| Delta 0.5Hz-4Hz (P4) | 19.88 (19.47, 20.22) | 19.94 (19.50, 20.22) | 0.507 |
| Theta 4Hz-8Hz (Fz) | 7.03 (6.77, 7.48) | 7.08 (6.74, 7.46) | 1.000 |
| Theta 4Hz-8Hz (F3) | 7.20 (6.93, 7.35) | 7.21 (6.84, 7.47) | 0.749 |
| Theta 4Hz-8Hz (F4) | 7.14 (6.86, 7.32) | 7.16 (6.90, 7.32) | 0.846 |
| Theta 4Hz-8Hz (Cz) | 7.11 (6.76, 7.30) | 7.14 (6.74, 7.37) | 0.681 |
| Theta 4Hz-8Hz (C3) | 7.78 (0.18)*^2^* | 7.79 (0.19) *^2^* | 0.936*^4^* |
| Theta 4Hz-8Hz (C4) | 7.60 (7.30, 7.71) | 7.53 (7.28, 7.71) | 0.378 |
| Theta 4Hz-8Hz (Pz) | 7.62 (7.45, 7.74) | 7.64 (7.45, 7.73) | 0.417 |
| Theta 4Hz-8Hz (P3) | 6.78 (6.68, 6.90) | 6.76 (6.55, 6.90) | 0.617 |
| Theta 4Hz-8Hz (P4) | 6.19 (0.27)*^2^* | 6.22 (0.30) *^2^* | 0.659*^4^* |
| Alpha 8Hz-12Hz (Fz) | 6.55 (6.11, 7.10) | 6.56 (6.11, 7.29) | 0.689 |
| Alpha 8Hz-12Hz (F3) | 5.66 (5.43, 5.92) | 5.66 (5.43, 6.13) | 0.516 |
| Alpha 8Hz-12Hz (F4) | 5.68 (5.43, 6.08) | 5.66 (5.40, 5.93) | 0.827 |
| Alpha 8Hz-12Hz (Cz) | 5.63 (5.43, 5.94) | 5.58 (5.43, 5.93) | 0.937 |
| Alpha 8Hz-12Hz (C3) | 5.93 (5.43, 7.74) | 6.11 (5.44, 7.71) | 0.480 |
| Alpha 8Hz-12Hz (C4) | 5.45 (5.22, 6.06) | 5.45 (5.34, 6.06) | 0.422 |
| Alpha 8Hz-12Hz (Pz) | 5.43 (5.29, 5.86) | 5.43 (5.29, 5.91) | 0.756 |
| Alpha 8Hz-12Hz (P3) | 5.93 (5.49, 7.02) | 6.13 (5.53, 7.03) | 0.526 |
| Alpha 8Hz-12Hz (P4) | 6.00 (5.43, 7.56) | 6.12 (5.49, 7.56) | 0.518 |
| SMR 12Hz-15Hz (Fz) | 5.43 (5.34, 5.50) | 5.48 (5.36, 5.50) | 0.469 |
| SMR 12Hz-15Hz (F3) | 5.13 (5.01, 5.44) | 5.31 (5.08, 5.67) | 0.376 |
| SMR 12Hz-15Hz (F4) | 5.37 (5.12, 5.65) | 5.37 (5.13, 5.69) | 0.455 |
| SMR 12Hz-15Hz (Cz) | 5.30 (5.16, 5.38) | 5.32 (5.16, 5.37) | 0.874 |
| SMR 12Hz-15Hz (C3) | 5.44 (5.16, 5.69) | 5.44 (5.28, 5.69) | 0.477 |
| SMR 12Hz-15Hz (C4) | 5.26 (5.06, 5.48) | 5.26 (5.06, 5.48) | 0.856 |
| SMR 12Hz-15Hz (Pz) | 5.58 (5.41, 5.73) | 5.58 (5.41, 5.73) | 0.690 |
| SMR 12Hz-15Hz (P3) | 5.29 (5.06, 5.45) | 5.29 (5.06, 5.45) | 0.874 |
| SMR 12Hz-15Hz (P4) | 4.56 (4.29, 6.45) | 4.56 (4.29, 6.47) | 0.635 |
| Beta-1 15Hz-20Hz (Fz) | 5.37 (5.11, 5.59) | 5.37 (5.11, 5.59) | 0.886 |
| Beta-1 15Hz-20Hz (F3) | 5.93 (5.78, 6.77) | 5.93 (5.84, 6.77) | 0.952 |
| Beta-1 15Hz-20Hz (F4) | 6.23 (6.07, 7.40) | 6.22 (6.08, 7.40) | 0.534 |
| Beta-1 15Hz-20Hz (Cz) | 5.81 (5.59, 6.59) | 5.81 (5.59, 6.59) | 0.528 |
| Beta-1 15Hz-20Hz (C3) | 5.72 (5.31, 6.34) | 5.86 (5.38, 6.34) | 0.777 |
| Beta-1 15Hz-20Hz (C4) | 5.69 (5.14, 7.14) | 6.07 (5.14, 7.14) | 0.534 |
| Beta-1 15Hz-20Hz (Pz) | 4.86 (4.43, 5.11) | 4.87 (4.44, 5.03) | 0.961 |
| Beta-1 15Hz-20Hz (P3) | 5.19 (5.00, 5.44) | 5.19 (5.00, 5.44) | 0.328 |
| Beta-1 15Hz-20Hz (P4) | 5.28 (4.93, 5.45) | 5.36 (4.93, 5.39) | 0.473 |
| Beta-2 20Hz-35Hz (Fz) | 4.09 (4.01, 4.21) | 4.09 (4.01, 4.31) | 0.353 |
| Beta-2 20Hz-35Hz (F3) | 3.96 (3.91, 4.09) | 3.97 (3.92, 4.09) | 0.785 |
| Beta-2 20Hz-35Hz (F4) | 4.35 (4.20, 4.89) | 4.51 (4.24, 4.88) | 0.586 |
| Beta-2 20Hz-35Hz (Cz) | 4.19 (4.08, 4.81) | 4.19 (4.08, 4.75) | 0.916 |
| Beta-2 20Hz-35Hz (C3) | 3.99 (3.86, 4.15) | 3.95 (3.86, 4.06) | 0.586 |
| Beta-2 20Hz-35Hz (C4) | 4.16 (4.02, 4.27) | 4.14 (4.07, 4.27) | 0.861 |
| Beta-2 20Hz-35Hz (Pz) | 4.32 (4.03, 4.97) | 4.32 (4.08, 4.97) | 0.708 |
| Beta-2 20Hz-35Hz (P3) | 4.56 (3.99, 4.76) | 4.56 (4.09, 4.62) | 0.875 |
| Beta-2 20Hz-35Hz (P4) | 4.56 (3.94, 4.67) | 4.56 (3.98, 4.64) | 0.948 |
| *^1^* *Mdn* (*Q1*, *Q3*)  *^2^* *M* (*SD*) | | | |
| *^3^* Wilcoxon rank-sum test with continuity correction  *^4^* Paired Welch's t-test | | | |

**Appendix D**. Effect of the intervention in the control group with eyes closed

| *QEEG parameter* | *Time point* | | *p-value^2^* |  |
| --- | --- | --- | --- | --- |
|  | *pre-fight,*  *n = 50^1^* | *post-fight,*  *n = 50^1^* |  |  |
| Delta 0.5Hz-4Hz (Fz) | 19.88 (19.08, 20.71) | 20.05 (19.09, 20.93) | 0.402 |  |
| Delta 0.5Hz-4Hz (F3) | 17.84 (16.04, 19.61) | 17.48 (16.01, 19.88) | 0.920 |  |
| Delta 0.5Hz-4Hz (F4) | 18.49 (17.90, 19.55) | 18.54 (18.11, 19.83) | 0.532 |  |
| Delta 0.5Hz-4Hz (Cz) | 19.46 (19.15, 19.84) | 19.49 (19.16, 19.93) | 0.464 |  |
| Delta 0.5Hz-4Hz (C3) | 19.25 (18.69, 19.83) | 19.25 (18.69, 19.97) | 0.424 |  |
| Delta 0.5Hz-4Hz (C4) | 19.30 (18.77, 19.90) | 19.40 (18.86, 19.98) | 0.402 |  |
| Delta 0.5Hz-4Hz (Pz) | 18.93 (18.44, 19.56) | 18.87 (18.36, 19.56) | 0.964 |  |
| Delta 0.5Hz-4Hz (P3) | 20.19 (19.59, 20.75) | 20.15 (19.47, 20.78) | 0.574 |  |
| Delta 0.5Hz-4Hz (P4) | 20.38 (19.71, 21.00) | 20.38 (19.65, 21.12) | 0.817 |  |
| Theta 4Hz-8Hz (Fz) | 11.45 (10.45, 12.95) | 11.45 (10.45, 12.87) | 0.942 |  |
| Theta 4Hz-8Hz (F3) | 11.74 (11.56, 12.26) | 11.76 (11.56, 12.26) | 0.534 |  |
| Theta 4Hz-8Hz (F4) | 10.72 (10.36, 11.96) | 10.54 (10.36, 11.91) | 0.368 |  |
| Theta 4Hz-8Hz (Cz) | 12.33 (10.90, 12.89) | 12.51 (11.54, 12.89) | 0.726 |  |
| Theta 4Hz-8Hz (C3) | 12.24 (9.59, 12.81) | 12.28 (11.06, 12.88) | 0.349 |  |
| Theta 4Hz-8Hz (C4) | 10.98 (8.92, 11.28) | 10.98 (8.92, 11.90) | 0.461 |  |
| Theta 4Hz-8Hz (Pz) | 11.32 (9.09, 12.28) | 11.36 (9.60, 12.34) | 0.655 |  |
| Theta 4Hz-8Hz (P3) | 10.12 (8.56, 10.23) | 10.23 (9.75, 10.23) | 0.550 |  |
| Theta 4Hz-8Hz (P4) | 11.10 (9.76, 11.44) | 11.20 (10.06, 11.54) | 0.355 |  |
| Alpha 8Hz-12Hz (Fz) | 6.87 (6.31, 7.84) | 6.87 (5.97, 7.91) | 0.952 |  |
| Alpha 8Hz-12Hz (F3) | 6.92 (6.13, 7.45) | 6.84 (6.13, 7.45) | 0.768 |  |
| Alpha 8Hz-12Hz (F4) | 6.88 (5.97, 7.29) | 6.88 (6.11, 7.29) | 0.523 |  |
| Alpha 8Hz-12Hz (Cz) | 6.03 (5.66, 7.53) | 6.03 (5.58, 7.53) | 0.601 |  |
| Alpha 8Hz-12Hz (C3) | 7.99 (7.92, 8.79) | 7.99 (6.13, 8.68) | 0.116 |  |
| Alpha 8Hz-12Hz (C4) | 7.14 (5.93, 9.43) | 6.13 (5.93, 7.18) | 0.164 |  |
| Alpha 8Hz-12Hz (Pz) | 6.18 (5.93, 8.68) | 6.13 (5.93, 8.27) | 0.336 |  |
| Alpha 8Hz-12Hz (P3) | 7.56 (6.18, 8.71) | 7.56 (6.13, 8.71) | 0.572 |  |
| Alpha 8Hz-12Hz (P4) | 7.56 (7.08, 7.75) | 7.56 (6.13, 7.59) | 0.147 |  |
| SMR 12Hz-15Hz (Fz) | 5.49 (5.43, 5.49) | 5.49 (5.43, 5.49) | 0.815 |  |
| SMR 12Hz-15Hz (F3) | 5.01 (5.01, 5.93) | 5.01 (5.01, 5.93) | 0.895 |  |
| SMR 12Hz-15Hz (F4) | 5.65 (5.28, 5.72) | 5.65 (5.28, 5.72) | 0.921 |  |
| SMR 12Hz-15Hz (Cz) | 5.32 (5.16, 5.47) | 5.32 (5.16, 5.47) | 0.970 |  |
| SMR 12Hz-15Hz (C3) | 5.38 (4.93, 5.69) | 5.38 (4.93, 5.69) | 0.873 |  |
| SMR 12Hz-15Hz (C4) | 5.26 (4.95, 5.48) | 5.26 (4.95, 5.48) | 0.895 |  |
| SMR 12Hz-15Hz (Pz) | 5.58 (5.37, 5.73) | 5.58 (5.37, 5.73) | 0.884 |  |
| SMR 12Hz-15Hz (P3) | 5.45 (5.29, 5.56) | 5.45 (5.29, 5.56) | 0.866 |  |
| SMR 12Hz-15Hz (P4) | 5.29 (4.99, 5.56) | 5.29 (4.99, 5.56) | 0.919 |  |
| Beta-1 15Hz-20Hz (Fz) | 5.47 (5.11, 5.59) | 5.57 (5.37, 5.62) | 0.648 |  |
| Beta-1 15Hz-20Hz (F3) | 5.92 (5.60, 6.77) | 5.92 (5.60, 6.70) | 0.795 |  |
| Beta-1 15Hz-20Hz (F4) | 6.21 (5.58, 6.40) | 6.21 (5.58, 6.40) | 0.399 |  |
| Beta-1 15Hz-20Hz (Cz) | 5.59 (5.20, 5.81) | 5.59 (5.20, 5.81) | 0.467 |  |
| Beta-1 15Hz-20Hz (C3) | 5.83 (5.39, 5.91) | 5.80 (5.39, 5.90) | **0.040** |  |
| Beta-1 15Hz-20Hz (C4) | 6.27 (5.03, 7.82) | 6.27 (5.03, 7.82) | 1.00 |  |
| Beta-1 15Hz-20Hz (Pz) | 4.51 (4.43, 5.43) | 4.51 (4.43, 5.34) | 0.577 |  |
| Beta-1 15Hz-20Hz (P3) | 5.89 (5.44, 6.05) | 5.89 (5.44, 6.05) | 0.658 |  |
| Beta-1 15Hz-20Hz (P4) | 4.84 (4.44, 5.43) | 4.84 (4.44, 5.36) | 0.194 |  |
| Beta-2 20Hz-35Hz (Fz) | 6.03 (5.31, 6.03) | 6.03 (5.48, 6.03) | 0.399 |  |
| Beta-2 20Hz-35Hz (F3) | 6.85 (6.74, 7.31) | 6.85 (6.74, 7.11) | 0.348 |  |
| Beta-2 20Hz-35Hz (F4) | 6.50 (6.45, 7.08) | 6.50 (6.45, 6.94) | 0.591 |  |
| Beta-2 20Hz-35Hz (Cz) | 5.93 (5.85, 5.93) | 5.93 (5.85, 5.93) | 0.896 |  |
| Beta-2 20Hz-35Hz (C3) | 5.67 (5.67, 6.40) | 5.67 (5.66, 6.22) | 0.516 |  |
| Beta-2 20Hz-35Hz (C4) | 5.73 (5.73, 6.69) | 5.73 (5.28, 6.63) | 0.495 |  |
| Beta-2 20Hz-35Hz (Pz) | 5.78 (5.53, 6.69) | 5.78 (5.53, 6.46) | 0.388 |  |
| Beta-2 20Hz-35Hz (P3) | 5.56 (5.38, 7.89) | 5.56 (5.38, 6.87) | 0.263 |  |
| Beta-2 20Hz-35Hz (P4) | 8.62 (7.46, 8.71) | 8.09 (7.46, 8.71) | 0.870 |  |
| *^1^* *Mdn* (*Q1*, *Q3*) | | | | |
| *^2^* Wilcoxon rank-sum test with continuity correction | | | | |

**Appendix E**. Pre-fight QEEG measurements between groups with eyes open

| *QEEG parameter* | *Group* | | *p-value^2^* |  |
| --- | --- | --- | --- | --- |
|  | *Control group, n = 50^1^* | *Experimental group, n = 50^1^* |  |  |
| Delta 0.5Hz-4Hz (Fz) | 19.9 (19.5, 20.2) | 20.1 (19.9, 20.8) | **0.001** |  |
| Delta 0.5Hz-4Hz (F3) | 21.5 (20.5, 22.7) | 22.0 (21.1, 23.8) | **0.016** |  |
| Delta 0.5Hz-4Hz (F4) | 19.9 (19.6, 20.5) | 21.5 (20.5, 22.6) | **<0.001** |  |
| Delta 0.5Hz-4Hz (Cz) | 17.4 (16.0, 18.6) | 14.3 (13.2, 15.6) | **<0.001** |  |
| Delta 0.5Hz-4Hz (C3) | 18.1 (16.3, 20.1) | 13.7 (13.3, 16.7) | **<0.001** |  |
| Delta 0.5Hz-4Hz (C4) | 16.6 (15.0, 18.3) | 13.6 (12.1, 17.7) | **<0.001** |  |
| Delta 0.5Hz-4Hz (Pz) | 18.1 (15.9, 19.7) | 11.5 (10.8, 12.6) | **<0.001** |  |
| Delta 0.5Hz-4Hz (P3) | 20.0 (19.6, 20.3) | 11.6 (11.2, 12.6) | **<0.001** |  |
| Delta 0.5Hz-4Hz (P4) | 19.9 (19.5, 20.2) | 9.2 (8.8, 9.9) | **<0.001** |  |
| Theta 4Hz-8Hz (Fz) | 7.0 (6.8, 7.5) | 12.4 (11.2, 14.8) | **<0.001** |  |
| Theta 4Hz-8Hz (F3) | 7.2 (6.9, 7.4) | 11.8 (10.8, 12.3) | **<0.001** |  |
| Theta 4Hz-8Hz (F4) | 7.1 (6.9, 7.3) | 12.1 (11.0, 13.9) | **<0.001** |  |
| Theta 4Hz-8Hz (Cz) | 7.1 (6.8, 7.3) | 7.4 (6.9, 8.5) | **<0.001** |  |
| Theta 4Hz-8Hz (C3) | 7.8 (7.6, 7.9) | 7.4 (6.8, 7.6) | **<0.001** |  |
| Theta 4Hz-8Hz (C4) | 7.6 (7.3, 7.7) | 6.6 (5.8, 7.0) | **<0.001** |  |
| Theta 4Hz-8Hz (Pz) | 7.6 (7.4, 7.7) | 6.5 (5.2, 7.0) | **<0.001** |  |
| Theta 4Hz-8Hz (P3) | 6.8 (6.7, 6.9) | 6.5 (6.0, 6.7) | **<0.001** |  |
| Theta 4Hz-8Hz (P4) | 6.2 (6.0, 6.4) | 5.2 (5.1, 6.1) | **<0.001** |  |
| Alpha 8Hz-12Hz (Fz) | 6.6 (6.1, 7.1) | 6.8 (6.4, 7.4) | **0.015** |  |
| Alpha 8Hz-12Hz (F3) | 5.7 (5.4, 5.9) | 6.6 (5.7, 7.0) | **<0.001** |  |
| Alpha 8Hz-12Hz (F4) | 5.7 (5.4, 6.1) | 9.5 (6.1, 11.5) | **<0.001** |  |
| Alpha 8Hz-12Hz (Cz) | 5.6 (5.4, 5.9) | 7.5 (5.7, 9.2) | **<0.001** |  |
| Alpha 8Hz-12Hz (C3) | 5.9 (5.4, 7.7) | 7.9 (5.7, 8.8) | **<0.001** |  |
| Alpha 8Hz-12Hz (C4) | 5.4 (5.2, 6.1) | 7.0 (4.9, 7.1) | 0.079 |  |
| Alpha 8Hz-12Hz (Pz) | 5.4 (5.3, 5.9) | 6.2 (5.3, 8.2) | **0.001** |  |
| Alpha 8Hz-12Hz (P3) | 5.9 (5.5, 7.0) | 7.8 (6.1, 8.7) | **0.001** |  |
| Alpha 8Hz-12Hz (P4) | 6.0 (5.4, 7.6) | 7.1 (4.8, 7.6) | 0.714 |  |
| SMR 12Hz-15Hz (Fz) | 5.4 (5.3, 5.5) | 4.0 (3.8, 4.6) | **<0.001** |  |
| SMR 12Hz-15Hz (F3) | 5.1 (5.0, 5.4) | 4.1 (3.8, 5.1) | **<0.001** |  |
| SMR 12Hz-15Hz (F4) | 5.4 (5.1, 5.7) | 5.1 (3.6, 5.2) | **<0.001** |  |
| SMR 12Hz-15Hz (Cz) | 5.3 (5.2, 5.4) | 4.2 (3.8, 5.2) | **<0.001** |  |
| SMR 12Hz-15Hz (C3) | 5.4 (5.2, 5.7) | 4.4 (3.8, 5.5) | **<0.001** |  |
| SMR 12Hz-15Hz (C4) | 5.3 (5.1, 5.5) | 4.4 (3.5, 4.9) | **<0.001** |  |
| SMR 12Hz-15Hz (Pz) | 5.6 (5.4, 5.7) | 4.9 (4.4, 5.8) | **<0.001** |  |
| SMR 12Hz-15Hz (P3) | 5.3 (5.1, 5.4) | 5.1 (4.4, 5.9) | 0.569 |  |
| SMR 12Hz-15Hz (P4) | 4.6 (4.3, 6.4) | 4.4 (3.6, 5.4) | **0.018** |  |
| Beta-1 15Hz-20Hz (Fz) | 5.4 (5.1, 5.6) | 5.0 (4.6, 5.1) | **<0.001** |  |
| Beta-1 15Hz-20Hz (F3) | 5.9 (5.8, 6.8) | 5.6 (5.0, 5.8) | **<0.001** |  |
| Beta-1 15Hz-20Hz (F4) | 6.2 (6.1, 7.4) | 6.4 (6.2, 7.3) | 0.247 |  |
| Beta-1 15Hz-20Hz (Cz) | 5.8 (5.6, 6.6) | 4.8 (4.6, 5.2) | **<0.001** |  |
| Beta-1 15Hz-20Hz (C3) | 5.7 (5.3, 6.3) | 5.4 (4.9, 5.8) | **0.003** |  |
| Beta-1 15Hz-20Hz (C4) | 5.7 (5.1, 7.1) | 5.0 (4.8, 5.3) | **<0.001** |  |
| Beta-1 15Hz-20Hz (Pz) | 4.9 (4.4, 5.1) | 4.8 (4.4, 5.4) | 0.530 |  |
| Beta-1 15Hz-20Hz (P3) | 5.2 (5.0, 5.4) | 5.4 (4.9, 5.9) | 0.767 |  |
| Beta-1 15Hz-20Hz (P4) | 5.3 (4.9, 5.4) | 5.0 (4.4, 5.4) | **0.047** |  |
| Beta-2 20Hz-35Hz (Fz) | 4.1 (4.0, 4.2) | 5.8 (5.2, 6.3) | **<0.001** |  |
| Beta-2 20Hz-35Hz (F3) | 4.0 (3.9, 4.1) | 7.8 (7.3, 8.5) | **<0.001** |  |
| Beta-2 20Hz-35Hz (F4) | 4.3 (4.2, 4.9) | 9.3 (7.1, 9.4) | **<0.001** |  |
| Beta-2 20Hz-35Hz (Cz) | 4.2 (4.1, 4.8) | 6.7 (6.2, 7.4) | **<0.001** |  |
| Beta-2 20Hz-35Hz (C3) | 4.0 (3.9, 4.2) | 6.4 (6.1, 7.2) | **<0.001** |  |
| Beta-2 20Hz-35Hz (C4) | 4.2 (4.0, 4.3) | 6.7 (6.4, 7.7) | **<0.001** |  |
| Beta-2 20Hz-35Hz (Pz) | 4.3 (4.0, 5.0) | 6.7 (6.4, 7.5) | **<0.001** |  |
| Beta-2 20Hz-35Hz (P3) | 4.6 (4.0, 4.8) | 7.1 (7.1, 8.1) | **<0.001** |  |
| Beta-2 20Hz-35Hz (P4) | 4.6 (3.9, 4.7) | 6.8 (6.1, 7.5) | **<0.001** |  |
| *^1^* *Mdn* (*Q1*, *Q3*) | | | | |
| *^2^* Test rank Wilcoxon | | | | |

**Appendix F**. Pre-fight QEEG measurements between groups with eyes closed

| *QEEG parameter* | *Group* | | *p-value^2^* |
| --- | --- | --- | --- |
|  | *Control group, n = 50^1^* | *Experimental group, n = 50^1^* |  |
| Delta 0.5Hz-4Hz (Fz) | 19.9 (19.1, 20.7) | 16.3 (13.8, 27.3) | **<0.001** |
| Delta 0.5Hz-4Hz (F3) | 17.8 (16.0, 19.6) | 12.8 (12.2, 14.2) | **<0.001** |
| Delta 0.5Hz-4Hz (F4) | 18.5 (17.9, 19.5) | 13.9 (12.3, 16.0) | **<0.001** |
| Delta 0.5Hz-4Hz (Cz) | 19.5 (19.2, 19.8) | 16.0 (15.0, 19.4) | **<0.001** |
| Delta 0.5Hz-4Hz (C3) | 19.2 (18.7, 19.8) | 14.2 (14.0, 16.3) | **<0.001** |
| Delta 0.5Hz-4Hz (C4) | 19.3 (18.8, 19.9) | 14.6 (13.3, 16.0) | **<0.001** |
| Delta 0.5Hz-4Hz (Pz) | 18.9 (18.4, 19.6) | 13.9 (12.3, 16.0) | **<0.001** |
| Delta 0.5Hz-4Hz (P3) | 20.2 (19.6, 20.7) | 13.0 (12.8, 19.6) | **<0.001** |
| Delta 0.5Hz-4Hz (P4) | 20.4 (19.7, 21.0) | 14.3 (13.3, 15.7) | **<0.001** |
| Theta 4Hz-8Hz (Fz) | 11.4 (10.4, 12.9) | 14.5 (11.8, 14.9) | **<0.001** |
| Theta 4Hz-8Hz (F3) | 11.7 (11.6, 12.3) | 12.3 (11.9, 14.1) | **0.001** |
| Theta 4Hz-8Hz (F4) | 10.7 (10.4, 12.0) | 13.9 (12.1, 14.7) | **<0.001** |
| Theta 4Hz-8Hz (Cz) | 12.3 (10.9, 12.9) | 8.5 (7.4, 9.8) | **<0.001** |
| Theta 4Hz-8Hz (C3) | 12.2 (9.6, 12.8) | 7.6 (6.6, 9.1) | **<0.001** |
| Theta 4Hz-8Hz (C4) | 11.0 (8.9, 11.3) | 6.7 (6.3, 10.4) | **<0.001** |
| Theta 4Hz-8Hz (Pz) | 11.3 (9.1, 12.3) | 9.7 (7.9, 10.6) | **<0.001** |
| Theta 4Hz-8Hz (P3) | 10.1 (8.6, 10.2) | 9.9 (8.0, 10.4) | 0.195 |
| Theta 4Hz-8Hz (P4) | 11.1 (9.8, 11.4) | 10.1 (8.2, 10.5) | **<0.001** |
| Alpha 8Hz-12Hz (Fz) | 6.9 (6.3, 7.8) | 8.6 (6.9, 9.5) | **<0.001** |
| Alpha 8Hz-12Hz (F3) | 6.9 (6.1, 7.4) | 7.4 (6.8, 9.7) | **0.003** |
| Alpha 8Hz-12Hz (F4) | 6.9 (6.0, 7.3) | 6.9 (6.1, 10.1) | 0.132 |
| Alpha 8Hz-12Hz (Cz) | 6.0 (5.7, 7.5) | 7.5 (5.7, 10.7) | 0.057 |
| Alpha 8Hz-12Hz (C3) | 8.0 (7.9, 8.8) | 8.8 (7.9, 9.7) | 0.232 |
| Alpha 8Hz-12Hz (C4) | 7.1 (5.9, 9.4) | 7.8 (7.2, 9.6) | **<0.001** |
| Alpha 8Hz-12Hz (Pz) | 6.2 (5.9, 8.7) | 8.7 (8.2, 11.0) | **<0.001** |
| Alpha 8Hz-12Hz (P3) | 7.6 (6.2, 8.7) | 8.7 (7.7, 11.7) | **0.001** |
| Alpha 8Hz-12Hz (P4) | 7.6 (7.1, 7.8) | 9.7 (7.6, 11.1) | **<0.001** |
| SMR 12Hz-15Hz (Fz) | 5.5 (5.4, 5.5) | 5.1 (4.4, 6.0) | **0.029** |
| SMR 12Hz-15Hz (F3) | 5.0 (5.0, 5.9) | 5.2 (4.1, 6.1) | 0.630 |
| SMR 12Hz-15Hz (F4) | 5.7 (5.3, 5.7) | 5.2 (5.1, 7.0) | 0.711 |
| SMR 12Hz-15Hz (Cz) | 5.3 (5.2, 5.5) | 5.4 (4.2, 5.9) | 0.827 |
| SMR 12Hz-15Hz (C3) | 5.4 (4.9, 5.7) | 4.6 (4.4, 5.2) | **<0.001** |
| SMR 12Hz-15Hz (C4) | 5.3 (5.0, 5.5) | 3.9 (3.7, 4.4) | **<0.001** |
| SMR 12Hz-15Hz (Pz) | 5.6 (5.4, 5.7) | 4.4 (4.0, 5.7) | **0.009** |
| SMR 12Hz-15Hz (P3) | 5.4 (5.3, 5.6) | 4.4 (4.4, 5.8) | **0.016** |
| SMR 12Hz-15Hz (P4) | 5.3 (5.0, 5.6) | 4.2 (3.6, 5.7) | **0.017** |
| Beta-1 15Hz-20Hz (Fz) | 5.5 (5.1, 5.6) | 5.0 (4.5, 5.1) | **<0.001** |
| Beta-1 15Hz-20Hz (F3) | 5.9 (5.6, 6.8) | 5.6 (5.0, 5.9) | **0.003** |
| Beta-1 15Hz-20Hz (F4) | 6.2 (5.6, 6.4) | 6.2 (5.6, 6.4) | 0.884 |
| Beta-1 15Hz-20Hz (Cz) | 5.6 (5.2, 5.8) | 6.2 (5.3, 6.4) | **0.023** |
| Beta-1 15Hz-20Hz (C3) | 5.8 (5.4, 5.9) | 6.4 (5.4, 6.9) | **0.037** |
| Beta-1 15Hz-20Hz (C4) | 6.3 (5.0, 7.8) | 5.4 (5.0, 6.3) | **0.036** |
| Beta-1 15Hz-20Hz (Pz) | 4.5 (4.4, 5.4) | 4.5 (4.4, 5.4) | 0.859 |
| Beta-1 15Hz-20Hz (P3) | 5.9 (5.4, 6.0) | 5.7 (5.0, 6.3) | 0.600 |
| Beta-1 15Hz-20Hz (P4) | 4.8 (4.4, 5.4) | 4.5 (4.4, 5.4) | 0.355 |
| Beta-2 20Hz-35Hz (Fz) | 6.0 (5.3, 6.0) | 5.7 (5.3, 6.3) | 0.989 |
| Beta-2 20Hz-35Hz (F3) | 6.8 (6.7, 7.3) | 7.8 (6.8, 8.5) | **0.013** |
| Beta-2 20Hz-35Hz (F4) | 6.5 (6.4, 7.1) | 8.2 (6.6, 9.4) | **0.001** |
| Beta-2 20Hz-35Hz (Cz) | 5.9 (5.8, 5.9) | 6.2 (5.5, 6.7) | 0.765 |
| Beta-2 20Hz-35Hz (C3) | 5.7 (5.7, 6.4) | 6.8 (6.4, 7.5) | **<0.001** |
| Beta-2 20Hz-35Hz (C4) | 5.7 (5.7, 6.7) | 7.4 (6.7, 7.9) | **<0.001** |
| Beta-2 20Hz-35Hz (Pz) | 5.8 (5.5, 6.7) | 7.5 (6.8, 7.8) | **<0.001** |
| Beta-2 20Hz-35Hz (P3) | 5.6 (5.4, 7.9) | 8.1 (7.1, 8.7) | **<0.001** |
| Beta-2 20Hz-35Hz (P4) | 8.6 (7.5, 8.7) | 8.6 (7.1, 8.7) | 0.948 |
| *^1^* *Mdn* (*Q1*, *Q3*)  *^2^* Test rank Wilcoxon | | | |

**Appendix G.** Post-fight QEEG measurements between groups with eyes open

| *QEEG parameter* | *Group* | | *p-value^2^* |
| --- | --- | --- | --- |
|  | *Control group, n = 50^1^* | *Experimental group, n = 50^1^* |  |
| Delta 0.5Hz-4Hz (Fz) | 19.9 (19.6, 20.2) | 29.0 (25.6, 32.9) | **<0.001** |
| Delta 0.5Hz-4Hz (F3) | 21.7 (20.4, 22.9) | 26.7 (25.5, 28.1) | **<0.001** |
| Delta 0.5Hz-4Hz (F4) | 20.1 (19.6, 20.6) | 26.1 (25.1, 26.6) | **<0.001** |
| Delta 0.5Hz-4Hz (Cz) | 16.6 (15.6, 18.4) | 15.6 (15.4, 19.2) | 0.370 |
| Delta 0.5Hz-4Hz (C3) | 18.1 (16.1, 20.1) | 13.8 (13.2, 16.5) | **<0.001** |
| Delta 0.5Hz-4Hz (C4) | 16.5 (14.9, 17.9) | 13.7 (13.1, 16.7) | **0.004** |
| Delta 0.5Hz-4Hz (Pz) | 18.3 (16.1, 20.2) | 37.0 (35.7, 38.8) | **<0.001** |
| Delta 0.5Hz-4Hz (P3) | 20.1 (19.6, 20.4) | 36.3 (31.7, 38.4) | **<0.001** |
| Delta 0.5Hz-4Hz (P4) | 19.9 (19.5, 20.2) | 36.9 (35.7, 37.9) | **<0.001** |
| Theta 4Hz-8Hz (Fz) | 7.1 (6.7, 7.5) | 15.8 (15.0, 17.0) | **<0.001** |
| Theta 4Hz-8Hz (F3) | 7.2 (6.8, 7.5) | 13.0 (10.9, 14.2) | **<0.001** |
| Theta 4Hz-8Hz (F4) | 7.2 (6.9, 7.3) | 13.7 (9.0, 16.9) | **<0.001** |
| Theta 4Hz-8Hz (Cz) | 7.1 (6.7, 7.4) | 8.6 (8.1, 9.0) | **<0.001** |
| Theta 4Hz-8Hz (C3) | 7.8 (7.7, 7.9) | 8.0 (7.8, 8.6) | **<0.001** |
| Theta 4Hz-8Hz (C4) | 7.5 (7.3, 7.7) | 7.2 (6.4, 8.2) | **0.010** |
| Theta 4Hz-8Hz (Pz) | 7.6 (7.4, 7.7) | 11.3 (8.0, 13.4) | **<0.001** |
| Theta 4Hz-8Hz (P3) | 6.8 (6.5, 6.9) | 10.4 (7.9, 13.5) | **<0.001** |
| Theta 4Hz-8Hz (P4) | 6.2 (6.0, 6.5) | 10.6 (6.8, 12.0) | **<0.001** |
| Alpha 8Hz-12Hz (Fz) | 6.6 (6.1, 7.3) | 7.2 (6.4, 8.1) | **0.006** |
| Alpha 8Hz-12Hz (F3) | 5.7 (5.4, 6.1) | 7.9 (6.4, 11.2) | **<0.001** |
| Alpha 8Hz-12Hz (F4) | 5.7 (5.4, 5.9) | 8.0 (5.5, 10.3) | **0.001** |
| Alpha 8Hz-12Hz (Cz) | 5.6 (5.4, 5.9) | 8.6 (5.7, 10.1) | **<0.001** |
| Alpha 8Hz-12Hz (C3) | 6.1 (5.4, 7.7) | 8.6 (6.3, 9.0) | **<0.001** |
| Alpha 8Hz-12Hz (C4) | 5.4 (5.3, 6.1) | 6.9 (5.2, 7.4) | **0.016** |
| Alpha 8Hz-12Hz (Pz) | 5.4 (5.3, 5.9) | 9.9 (9.5, 12.6) | **<0.001** |
| Alpha 8Hz-12Hz (P3) | 6.1 (5.5, 7.0) | 8.0 (7.9, 9.0) | **<0.001** |
| Alpha 8Hz-12Hz (P4) | 6.1 (5.5, 7.6) | 6.6 (6.4, 8.4) | **0.009** |
| SMR 12Hz-15Hz (Fz) | 5.5 (5.4, 5.5) | 4.9 (4.3, 5.5) | **<0.001** |
| SMR 12Hz-15Hz (F3) | 5.3 (5.1, 5.7) | 6.0 (5.3, 7.0) | **<0.001** |
| SMR 12Hz-15Hz (F4) | 5.4 (5.1, 5.7) | 5.3 (4.2, 6.3) | 0.738 |
| SMR 12Hz-15Hz (Cz) | 5.3 (5.2, 5.4) | 5.5 (4.5, 6.2) | **0.046** |
| SMR 12Hz-15Hz (C3) | 5.4 (5.3, 5.7) | 6.0 (4.8, 7.2) | 0.057 |
| SMR 12Hz-15Hz (C4) | 5.3 (5.1, 5.5) | 4.6 (4.2, 5.2) | **<0.001** |
| SMR 12Hz-15Hz (Pz) | 5.6 (5.4, 5.7) | 7.3 (6.1, 8.9) | **<0.001** |
| SMR 12Hz-15Hz (P3) | 5.3 (5.1, 5.4) | 5.6 (4.8, 6.8) | 0.350 |
| SMR 12Hz-15Hz (P4) | 4.6 (4.3, 6.5) | 5.2 (4.4, 6.5) | 0.402 |
| Beta-1 15Hz-20Hz (Fz) | 5.4 (5.1, 5.6) | 5.6 (5.0, 5.7) | 0.959 |
| Beta-1 15Hz-20Hz (F3) | 5.9 (5.8, 6.8) | 6.6 (5.6, 7.3) | 0.251 |
| Beta-1 15Hz-20Hz (F4) | 6.2 (6.1, 7.4) | 6.7 (6.3, 7.0) | 0.656 |
| Beta-1 15Hz-20Hz (Cz) | 5.8 (5.6, 6.6) | 6.0 (5.4, 6.6) | 0.970 |
| Beta-1 15Hz-20Hz (C3) | 5.9 (5.4, 6.3) | 6.2 (5.6, 7.1) | 0.072 |
| Beta-1 15Hz-20Hz (C4) | 6.1 (5.1, 7.1) | 5.2 (4.4, 5.7) | **0.001** |
| Beta-1 15Hz-20Hz (Pz) | 4.9 (4.4, 5.0) | 6.3 (5.7, 7.7) | **<0.001** |
| Beta-1 15Hz-20Hz (P3) | 5.2 (5.0, 5.4) | 6.5 (6.0, 7.0) | **<0.001** |
| Beta-1 15Hz-20Hz (P4) | 5.4 (4.9, 5.4) | 5.8 (5.2, 7.1) | **0.001** |
| Beta-2 20Hz-35Hz (Fz) | 4.1 (4.0, 4.3) | 6.4 (5.7, 8.1) | **<0.001** |
| Beta-2 20Hz-35Hz (F3) | 4.0 (3.9, 4.1) | 9.8 (7.6, 11.4) | **<0.001** |
| Beta-2 20Hz-35Hz (F4) | 4.5 (4.2, 4.9) | 9.5 (7.4, 10.5) | **<0.001** |
| Beta-2 20Hz-35Hz (Cz) | 4.2 (4.1, 4.8) | 6.9 (6.6, 8.1) | **<0.001** |
| Beta-2 20Hz-35Hz (C3) | 3.9 (3.9, 4.1) | 7.9 (6.7, 8.3) | **<0.001** |
| Beta-2 20Hz-35Hz (C4) | 4.1 (4.1, 4.3) | 6.7 (5.7, 10.1) | **<0.001** |
| Beta-2 20Hz-35Hz (Pz) | 4.3 (4.1, 5.0) | 8.1 (7.1, 9.5) | **<0.001** |
| Beta-2 20Hz-35Hz (P3) | 4.6 (4.1, 4.6) | 8.4 (8.1, 9.8) | **<0.001** |
| Beta-2 20Hz-35Hz (P4) | 4.6 (4.0, 4.6) | 9.1 (8.4, 9.1) | **<0.001** |
| *^1^* *Mdn* (*Q1*, *Q3*)  *^2^* Test rank Wilcoxon | | | |

**Appendix H.** Post-fight QEEG measurements between groups with eyes closed

| QEEG parameter | Group | | p-value^2^ |
| --- | --- | --- | --- |
|  | Control group, N = 50^1^ | Experimental group, N = 50^1^ |  |
| Delta 0.5Hz-4Hz (Fz) | 20.0 (19.1, 20.9) | 20.8 (17.7, 24.0) | 0.697 |
| Delta 0.5Hz-4Hz (F3) | 17.5 (16.0, 19.9) | 22.3 (13.4, 32.7) | 0.231 |
| Delta 0.5Hz-4Hz (F4) | 18.5 (18.1, 19.8) | 21.0 (12.2, 28.3) | 0.661 |
| Delta 0.5Hz-4Hz (Cz) | 19.5 (19.2, 19.9) | 14.9 (13.4, 15.7) | <0.001 |
| Delta 0.5Hz-4Hz (C3) | 19.2 (18.7, 20.0) | 16.4 (13.0, 17.4) | <0.001 |
| Delta 0.5Hz-4Hz (C4) | 19.4 (18.9, 20.0) | 12.6 (11.1, 17.1) | <0.001 |
| Delta 0.5Hz-4Hz (Pz) | 18.9 (18.4, 19.6) | 21.3 (19.3, 29.8) | 0.001 |
| Delta 0.5Hz-4Hz (P3) | 20.1 (19.5, 20.8) | 25.9 (21.3, 26.7) | <0.001 |
| Delta 0.5Hz-4Hz (P4) | 20.4 (19.6, 21.1) | 25.7 (18.2, 26.6) | 0.006 |
| Theta 4Hz-8Hz (Fz) | 11.4 (10.4, 12.9) | 11.8 (9.2, 26.7) | 0.844 |
| Theta 4Hz-8Hz (F3) | 11.8 (11.6, 12.3) | 16.8 (12.9, 17.6) | <0.001 |
| Theta 4Hz-8Hz (F4) | 10.5 (10.4, 11.9) | 16.9 (12.1, 17.7) | <0.001 |
| Theta 4Hz-8Hz (Cz) | 12.5 (11.5, 12.9) | 9.0 (7.3, 10.2) | <0.001 |
| Theta 4Hz-8Hz (C3) | 12.3 (11.1, 12.9) | 8.0 (6.9, 9.6) | <0.001 |
| Theta 4Hz-8Hz (C4) | 11.0 (8.9, 11.9) | 6.7 (5.9, 7.5) | <0.001 |
| Theta 4Hz-8Hz (Pz) | 11.4 (9.6, 12.3) | 10.2 (9.1, 10.9) | 0.006 |
| Theta 4Hz-8Hz (P3) | 10.2 (9.8, 10.2) | 10.6 (9.6, 11.5) | 0.006 |
| Theta 4Hz-8Hz (P4) | 11.2 (10.1, 11.5) | 7.8 (6.4, 8.8) | <0.001 |
| Alpha 8Hz-12Hz (Fz) | 6.9 (6.0, 7.9) | 11.9 (8.4, 12.9) | <0.001 |
| Alpha 8Hz-12Hz (F3) | 6.8 (6.1, 7.4) | 14.2 (8.1, 19.5) | <0.001 |
| Alpha 8Hz-12Hz (F4) | 6.9 (6.1, 7.3) | 14.0 (6.7, 21.1) | <0.001 |
| Alpha 8Hz-12Hz (Cz) | 6.0 (5.6, 7.5) | 14.8 (9.2, 19.3) | <0.001 |
| Alpha 8Hz-12Hz (C3) | 8.0 (6.1, 8.7) | 11.4 (8.0, 15.5) | <0.001 |
| Alpha 8Hz-12Hz (C4) | 6.1 (5.9, 7.2) | 9.5 (7.2, 11.7) | <0.001 |
| Alpha 8Hz-12Hz (Pz) | 6.1 (5.9, 8.3) | 12.0 (10.1, 13.6) | <0.001 |
| Alpha 8Hz-12Hz (P3) | 7.6 (6.1, 8.7) | 10.8 (8.9, 13.8) | <0.001 |
| Alpha 8Hz-12Hz (P4) | 7.6 (6.1, 7.6) | 10.8 (7.2, 11.6) | <0.001 |
| SMR 12Hz-15Hz (Fz) | 5.5 (5.4, 5.5) | 5.8 (4.9, 5.8) | 0.115 |
| SMR 12Hz-15Hz (F3) | 5.0 (5.0, 5.9) | 6.5 (5.7, 7.5) | <0.001 |
| SMR 12Hz-15Hz (F4) | 5.7 (5.3, 5.7) | 5.8 (5.1, 6.7) | 0.046 |
| SMR 12Hz-15Hz (Cz) | 5.3 (5.2, 5.5) | 5.7 (5.1, 7.2) | 0.011 |
| SMR 12Hz-15Hz (C3) | 5.4 (4.9, 5.7) | 6.4 (5.4, 7.8) | <0.001 |
| SMR 12Hz-15Hz (C4) | 5.3 (5.0, 5.5) | 5.3 (4.0, 6.2) | 0.811 |
| SMR 12Hz-15Hz (Pz) | 5.6 (5.4, 5.7) | 5.7 (5.0, 7.2) | 0.106 |
| SMR 12Hz-15Hz (P3) | 5.4 (5.3, 5.6) | 5.3 (4.9, 8.9) | 0.790 |
| SMR 12Hz-15Hz (P4) | 5.3 (5.0, 5.6) | 5.0 (4.6, 7.6) | 0.882 |
| Beta-1 15Hz-20Hz (Fz) | 5.6 (5.4, 5.6) | 6.1 (5.3, 6.8) | 0.005 |
| Beta-1 15Hz-20Hz (F3) | 5.9 (5.6, 6.7) | 6.7 (5.9, 7.1) | <0.001 |
| Beta-1 15Hz-20Hz (F4) | 6.2 (5.6, 6.4) | 6.1 (5.9, 6.4) | 0.190 |
| Beta-1 15Hz-20Hz (Cz) | 5.6 (5.2, 5.8) | 6.4 (5.5, 7.3) | <0.001 |
| Beta-1 15Hz-20Hz (C3) | 5.8 (5.4, 5.9) | 6.6 (6.3, 8.5) | <0.001 |
| Beta-1 15Hz-20Hz (C4) | 6.3 (5.0, 7.8) | 5.7 (5.4, 5.8) | 0.564 |
| Beta-1 15Hz-20Hz (Pz) | 4.5 (4.4, 5.3) | 6.8 (5.8, 7.8) | <0.001 |
| Beta-1 15Hz-20Hz (P3) | 5.9 (5.4, 6.0) | 6.2 (5.8, 6.5) | 0.012 |
| Beta-1 15Hz-20Hz (P4) | 4.8 (4.4, 5.4) | 5.6 (5.2, 6.3) | <0.001 |
| Beta-2 20Hz-35Hz (Fz) | 6.0 (5.5, 6.0) | 6.7 (5.9, 7.2) | <0.001 |
| Beta-2 20Hz-35Hz (F3) | 6.8 (6.7, 7.1) | 7.2 (6.9, 7.8) | 0.001 |
| Beta-2 20Hz-35Hz (F4) | 6.5 (6.4, 6.9) | 7.0 (6.2, 8.4) | 0.552 |
| Beta-2 20Hz-35Hz (Cz) | 5.9 (5.9, 5.9) | 7.3 (6.4, 7.8) | <0.001 |
| Beta-2 20Hz-35Hz (C3) | 5.7 (5.7, 6.2) | 7.1 (6.7, 8.3) | <0.001 |
| Beta-2 20Hz-35Hz (C4) | 5.7 (5.3, 6.6) | 6.8 (6.6, 7.9) | <0.001 |
| Beta-2 20Hz-35Hz (Pz) | 5.8 (5.5, 6.5) | 7.7 (7.1, 9.1) | <0.001 |
| Beta-2 20Hz-35Hz (P3) | 5.6 (5.4, 6.9) | 7.9 (7.5, 8.7) | <0.001 |
| Beta-2 20Hz-35Hz (P4) | 8.1 (7.5, 8.7) | 7.0 (6.2, 8.6) | 0.011 |
| ^1^ *Mdn* (Q1, Q3)  ^2^ Test rank Wilcoxon | | | |

**Appendix I.** Effect of direct head blows on QEEG measurements

| *QEEG parameter* | *Direct head blows* | |
| --- | --- | --- |
|  | *r* | *p* |
| Delta 0.5Hz-4Hz (Fz) | 0.36 | **0.011** |
| Delta 0.5Hz-4Hz (C3) | 0.38 | **0.006** |
| Delta 0.5Hz-4Hz (C4) | 0.47 | **0.001** |
| Delta 0.5Hz-4Hz (Pz) | 0.43 | **0.002** |
| Delta 0.5Hz-4Hz (P3) | 0.50 | **<0.001** |
| Delta 0.5Hz-4Hz (P4) | 0.55 | **<0.001** |
| Beta-2 20Hz-35Hz (Cz) | 0.32 | **0.022** |
